# Learning from Nature: Bio-Inspired Designs and Strategies for Efficient On-Earth and Off-Earth Ventilation Systems

**DOI:** 10.3390/biomimetics10110754

**Published:** 2025-11-07

**Authors:** Ulfa Riani, Noune Melkoumian, David Harvey, Rini Akmeliawati

**Affiliations:** 1 Discipline of Mining and Petroleum Engineering, School of Chemical Engineering, The University of Adelaide, Adelaide 5005, Australia; noune.melkoumian@adelaide.edu.au; 2 School of Electrical and Mechanical Engineering, The University of Adelaide, Adelaide 5005, Australia; david.harvey@adelaide.edu.au (D.H.)

**Keywords:** bio-inspired ventilation, reduced noise fans, adaptive ventilation systems, ventilation systems for off-Earth habitats, mine ventilation systems

## Abstract

Efficient ventilation systems are of paramount importance for maintaining optimal air quality in indoor and enclosed environments, both on Earth and in space. Such environments include buildings, space habitats, international space station crew quarters, tunnels, underground mines and other structures. However, conventional ventilation systems encounter various challenges, including uneven air distribution, energy inefficiency, noise, and limited adaptability to fluctuating environmental conditions. Concurrently, a multitude of organisms in nature have demonstrated the capacity to construct structures that can facilitate efficient air exchange and heat regulation. Illustrative examples of such structures include ant nests, termite mounds and prairie dog burrows. The present study explores, analyses and summarizes the mechanisms, structures and strategies found in nature that can inspire the design of efficient and effective ventilation systems. The purpose of this paper is to highlight the practical implications of the aforementioned designs. To this end, it reviews the progress of research into bio-inspired ventilation, focusing on the following three areas: air regulation, component optimization and environmentally adaptive strategies. A bibliometric analysis and research trend is presented to illustrate the key developments in this field over the past 25 years. The potential of integrating the bio-inspired strategies into ventilation systems, with a particular emphasis on off-Earth habitats and underground mines, is discussed. This study provides a comprehensive overview of the development of bio-inspired ventilation systems, thereby establishing the foundation for the creation of innovative and efficient design solutions.

## 1. Introduction

On-Earth ventilation systems, which are often an integral part of the heating, ventilation and air-conditioning (HVAC) systems, commonly face issues associated mainly with the energy consumption and efficiency. HVAC systems contribute approximately 40% to the building’s energy consumption globally [1] and almost 50% in hot and arid regions [2]. They also account for around 40% of the primary energy use in the United States, with China projected to reach a similar level in the coming years [3]. While in some cases natural ventilation can accommodate the supply of fresh air to buildings on Earth, it is often not capable of providing sufficient cooling to the incoming air during the high-temperature seasons, which results in thermal discomfort for the occupants [4]. Consequently, a significant percentage of modern buildings are still highly dependent on mechanical ventilation systems, which are energy-intensive and take up a considerable amount of space due to their complexity and large volume [5].

A similar but far more critical challenge exists in the mining industry. While the fundamental objective of a mine ventilation system appears straightforward, which is to deliver a sufficient air quantity and quality to all areas of the mine where personnel are present, the reality has become increasingly complex [6]. As easily accessible mineral reserves become depleted, the mining operations are forced to go deeper underground, where conditions are more hostile, hotter and gassier [7,8,9,10,11]. At greater depths, the ventilation system must not only supply fresh air but also address a range of compounding risks, including the serious threat of heat stress to workers and equipment, the increased likelihood of mine fires, the presence of explosive dust and gases, and the growing use of mechanized diesel-powered machinery, which generates more heat and emits toxic exhaust. Beyond safety, there is also the matter of economics, where ventilation systems generally account for a significant portion of the mine’s operating energy costs [10]. The combination of these factors creates a pressing need for optimizing mine ventilation systems so that they can effectively balance safety, health, and operational efficiency while minimizing energy consumption.

As space exploration advances and the focus shifts toward longer and more sustained crewed missions, the next frontier in ventilation challenges lies beyond the Earth itself, where future off-Earth habitats will depend on effective systems to maintain safe and optimal indoor climates [12]. Unlike on Earth, where the natural convection driven by the temperature and density differences helps the movement of the air, the microgravity conditions of space eliminate this process [13,14]. Consequently, without an adequate forced airflow, air with a high CO_2_ concentration from breathing tends to accumulate around the head of astronauts when they are in stationary positions such as sleeping [15,16], which could lead to various health risks, such as headaches and asphyxiation [17]. A prominent real-world example of ventilation in space habitats is aboard the International Space Station (ISS). Although the general ventilation systems on the ISS are sufficient to accommodate most situations, this might not be the case for the astronauts’ sleeping quarters or crew quarters (CQ) [13,18,19]. According to several NASA and ESA reports, the crew members frequently wake up with symptoms of CO_2_ intoxication due to the localized build-up of CO_2_ around their heads, while the temperatures in CQs often exceed the standard for comfortable levels [20]. The ISS environment also becomes increasingly polluted with dust and particulate matter, primarily originating from the occupants themselves. As dust travels through the ducting of the ventilation systems, it can cause issues with the airflow sensors monitoring the flow variations [19]. Additionally, as much as 13% of the CQs’ total mass and 6% of its volume are dedicated to noise reduction measures. Unlike the ISS, which is located in low Earth orbit, this allocation of mass and volume specifically for noise reduction in the ventilation system could be impractical for deeper space missions beyond the current ISS [20].

With the need to provide effective and reliable life support systems for future long-duration crewed space missions [21,22] and the growing demand to reduce the energy consumption as well as to optimize the ventilation systems of buildings and mines on Earth [2,23], developing an innovative, energy-efficient, and effective nature-inspired ventilation system could provide a sound solution to these challenges. For more than 3.8 billion years, many organisms in nature have evolved to be sustainable, efficient and adaptable. They are capable of self-regulation through feedback mechanisms, resilient to sudden changes and adaptable to new conditions [24]. Like the built environment, organisms in nature require effective air regulation and heat transfer mechanisms because sufficient oxygen and thermal comfort are vital for their survival. In the past 25 years, bio-inspiration has gained significant attention across various fields of research [25], including the built environment, where researchers have adopted design principles found in nature to improve the performance of buildings [26,27].

Previous reviews on bio-inspired designs and technologies in the built environment have focused largely on the thermal regulation of buildings, including cooling strategies [28], energy-efficient built environment [29], thermal energy regulation [30], and applications of thermoregulation strategies in the architecture [31]. While these works provide valuable insights into improving the energy performance of buildings, the discussions specifically addressing the ventilation systems are often limited. Moreover, these studies lack a focused discussion of the research trends in bio-inspired ventilation and its components over the past decade, which would reveal how the field has evolved in terms of design strategies, modelling approaches, application contexts, and performance outcomes. They overlook both the progress already achieved and the potential applications of the bio-inspired ventilation in the critical contexts such as underground mines and off-Earth habitats.

The primary aim of this paper is to provide a comprehensive overview and critical evaluation of bio-inspired engineering features, structures, systems, and strategies that are relevant to the design and improvement of ventilation systems and their components. It investigates and discusses how these biological designs and principles have been applied and tested across different contexts, highlighting the advantages and limitations of each approach. A bibliometric and research trend analysis is also included to examine how the field of bio-inspired ventilation has evolved over the past 25 years in terms of publication growth, disciplinary distribution, research focus, and geographical contributions. Finally, the paper explores the potential applications and integration of bio-inspired ventilation strategies in critical environments such as space habitats and underground mines, where existing systems face significant environmental challenges. Section 1 of the paper introduces the background and motivation for the study and outlines the research problem and objectives. Section 2 describes the methodology for identifying and reviewing the relevant literature, along with the research trend analysis. Section 3, Section 4 and Section 5 discuss the various insights and inspiration from nature for designing effective and efficient ventilation systems and their components. Section 6 presents potential applications and integration of such designs and principles for off-Earth habitats and on-Earth underground mines, along with associated challenges. Section 7 presents research gaps and recommendations for future research. Section 8 concludes the findings.

## 2. Methodology

**Figure 1:**
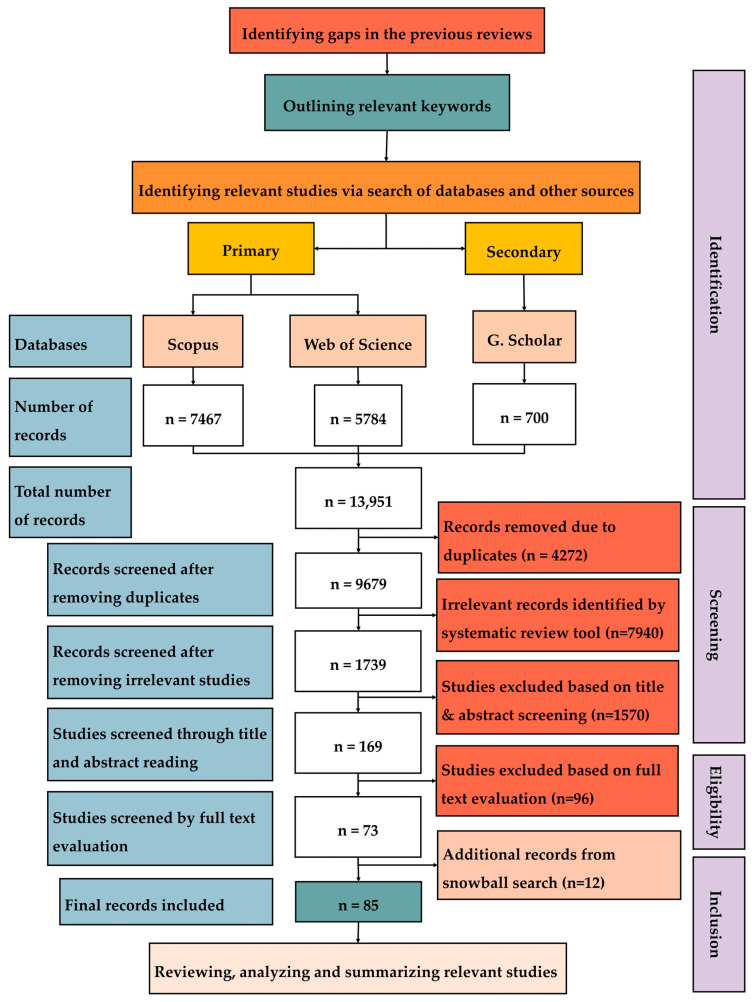
The workflow and search process of the presented review developed based on the PRISMA approach.

This review has been conducted according to the preferred reporting items for systematic reviews and meta-analyses (PRISMA) guidelines, comprising four key stages: (1) identifying studies through database searches and other sources, (2) screening the studies to select the relevant literature, (3) assessing the eligibility of full-text articles based on specific inclusion criteria, and (4) including the final set of studies for review. Figure 1 illustrates the detailed process of this study, from identifying the gaps in the existing literature to finding, reviewing, analyzing, and summarizing (synthesizing) the relevant studies. The gap identification process began with a review of the existing literature and systematic reviews on bio-inspired design and ventilation systems to determine the extent to which ventilation-specific aspects have been addressed. Based on these findings, a comprehensive search strategy was developed to capture relevant studies across multiple databases and disciplines. The next stages of this review, including data identification, screening, eligibility, and inclusion, along with their respective processes, as illustrated in Figure 1, are presented and discussed in detail in the following subsections.

### 2.1. Data Identification

To ensure that all the relevant literature was captured, the document identification process began with outlining the relevant keywords, which were then used to formulate specific search queries, as presented in Table 1. Keywords such as “bio-inspired” and its various synonyms, e.g., biomimetic, nature-inspired, bio-inspiration, nature-based, biomimicry, bio-design, bionic, organism-inspired and plant-inspired, were used. These biomimicry-related keywords were combined with the terms related to ventilation systems, their components and the built environment, such as vent, duct, fan, air regulation, wall, envelope, air circulation and HVAC systems. Boolean operators (AND/OR) were employed to refine the search. For the initial search in Scopus and Web of Science, to cover all relevant terms comprehensively, an asterisk (*) was added at the end of the subject-related words. For instance, using “vent*” ensures that variations like vents, ventilation, ventilating, and venting are included. To cover the progress of the research over the past 25 years, the results were limited to documents that have been published between 2000 and 2025 inclusive. Scopus and Web of Science were selected as the primary database sources due to their comprehensive coverage of peer-reviewed research. To prevent the potential biases introduced by Google Scholar’s proprietary ranking algorithm, it was utilized only as a secondary data source to identify additional studies not indexed in the primary databases. For the literature search in Google Scholar, the advanced search tool was utilized using keywords similar to those used in Scopus and Web of Science. Only the first 100 articles returned by Google Scholar for each search query were selected for further screening, because the documents beyond this were generally found to be irrelevant to this review. The total number of documents identified from Scopus, Web of Science, and Google Scholar was 7467, 5784, and 700, respectively. In addition, the reference lists of papers that passed the full-text evaluation were manually checked to identify any relevant studies that may have been missed during the initial database searches. The documents obtained through this process were categorized under the snowball search method, which yielded an additional 12 documents.

### 2.2. Data Screening and Inclusion

As shown in Figure 1, the total number of documents retrieved from multiple databases was 13,951, covering a wide range of subject areas, which are discussed in detail under Section 2.3. To refine the initial search results and to exclude irrelevant documents for this review, a systematic review tool, namely Rayyan, was used before proceeding with the manual screening through abstract reading and full-text eligibility checks. This tool helps in identifying and removing duplicate entries due to the overlap of similar records across different databases. To further remove irrelevant documents, the exclusion filter in Rayyan was used to exclude the literature with keywords outside of the scope of ventilation systems and the built environment, e.g., signal processing, peptides, bacteria, propulsion, and robots. The duplicate removal process reduced the total number of documents to 9679, while screening for irrelevant studies brought this down to 1379. After reviewing titles and abstracts, this was further reduced to 169 documents. At the final stage of the data collection, which involved a full-text evaluation or eligibility check, 73 documents were identified. Additionally, 12 more documents were obtained through a snowball search. The final number of documents included for analysis is 85. The inclusion and exclusion criteria used for this review are summarized in Table 2.

### 2.3. Data Analysis

After the data inclusion stage, the next step in this review is the analysis and synthesis of the relevant studies. In this section, a bibliometric and comparative analysis of the data is presented, while the detailed discussion and synthesis of the selected studies are provided in Section 3, Section 4 and Section 5. Bibliometric analysis is a systematic research method that employs statistical and mathematical tools to quantitatively and structurally examine large volumes of academic data. It utilizes publication metadata such as authors, institutions, countries, keywords, and topics to reveal how a research field has evolved over time, to highlight emerging areas of investigation, and to identify patterns in the structural and collaborative relationships among research constituents [32].

#### 2.3.1. Research Trend and Bibliometric Analysis

**Figure 2:**
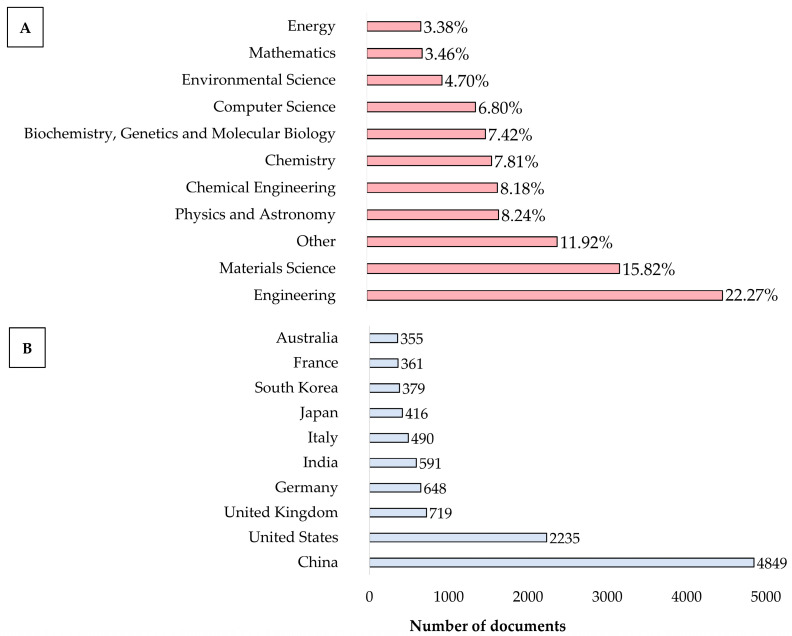
(**A**) Percentage of published documents categorized by different focus areas before screening; (**B**) number of published documents by the top 10 most productive countries.

**Figure 3:**
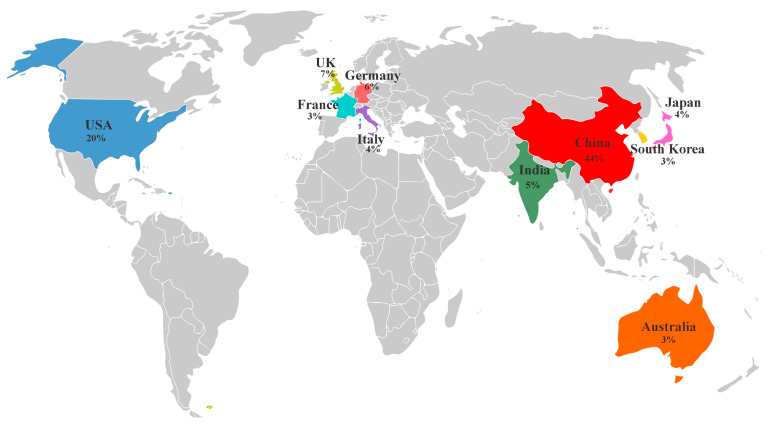
World map showing the percentage of scientific output of the top 10 most productive countries in the field of bio-inspired ventilation systems and the built environment before the screening process. Adapted from [33], public domain.

Although the pre-screened dataset comprises a large number of documents, examining its trends is crucial for understanding the impact of bio-inspiration in the engineering fields and related sciences. Such analysis not only highlights the growth and evolution of the field but also identifies the shifts in the research focus and emerging areas. The thematic analysis of the pre-screened records revealed that engineering constituted the largest segment at 22.3%, followed by materials science at 15.8%, physics and astronomy at 8.24%, chemical engineering at 8.18%, and chemistry at 7.8%, as shown in Figure 2A. The “others” category, accounting for 11.9%, includes additional disciplines such as medicine, social sciences, biological sciences, and agriculture. This diversity in subject areas is likely due to the widespread use of terms such as bio-inspired, vent, cooling, heating, and wall across multiple disciplines. Hence, this underscores the importance of further screening to ensure the inclusion of the most relevant articles for further analysis. In terms of the most productive countries, China and the United States lead the trend in the number of publications, contributing approximately 4800 and 2200 documents, respectively, as shown in Figure 2B. Several European countries are also among the top 10 most productive, including the United Kingdom, Germany, Italy and France. Meanwhile, in Asia, the most productive countries, in addition to China, are India, South Korea, and Japan. Australia is also among the leading contributors, ranking 10th in the trend. Figure 3 shows the scientific production of the ten most productive countries, expressed in percentages.

**Figure 4:**
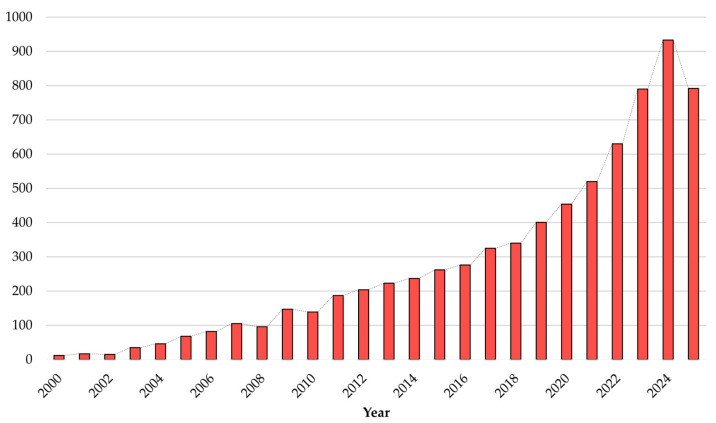
Number of published documents categorized by year.

In addition to classifying the retrieved documents into various subject areas and countries, this study also examined the number of publications over the years. As shown in Figure 4, from 2000 to 2010, the number of published documents remained relatively low but exhibited an overall upward trend, despite some fluctuations. This trend demonstrates the growing interest in the field, along with the possibility of competing paradigms in its early stages, leading to fluctuations in the number of publications. From 2010 to 2017, there was a more noticeable increase in publication numbers compared to the previous decade, reflecting a sustained interest in this field of research. The most significant surge occurred between 2018 and 2025, during which the number of publications rose sharply, accounting for more than half of the total publications in the last 25 years. The surge is likely driven by the rising demand for more sustainable buildings and energy-efficient systems over the last few years. For 2025, although the data are still incomplete for the current year, the total number of publications has already surpassed that of 2023 and earlier years, indicating that interest in this field is likely to continue growing.

**Figure 5:**
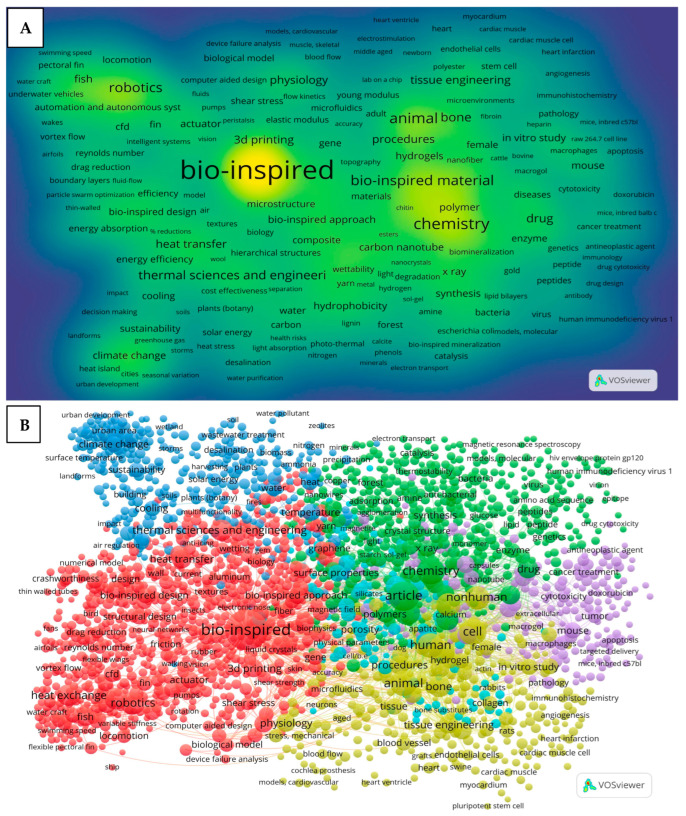
Keyword co-occurrence maps before the screening process: (**A**) density visualization; (**B**) network visualization.

To further investigate the research trends, a keyword co-occurrence analysis was conducted on the dataset before and after the screening process using VOSviewer software (version 1.6.20). Two types of keyword co-occurrence visualizations are presented in this study: network visualization and density visualization. The density visualization highlights areas of high and low keyword occurrence across the dataset using a color gradient ranging from blue to green to yellow. A color closer to yellow indicates a more frequently appearing keyword, whereas a color closer to blue indicates a keyword that is mentioned less frequently. Meanwhile, the network visualization map is used to display the relationships between keywords as a network of nodes and links. The lines between the nodes indicate the strength of the co-occurrence relationships, with shorter distances between the nodes representing stronger connections or closer associations between the keywords. Before the screening process, even with an occurrence threshold of 20, the dataset still produced 1640 terms due to the high volume of the records analyzed. As shown in Figure 5A, the most frequent keywords were bio-inspired, chemistry, bio-inspired material, robotics, and animal bone. Many of these terms are either unrelated to ventilation systems or are too broad in scope. Also, the relationships between these keywords were unclear (Figure 5B), as the excessive number of terms led to dense interconnections. To allow for a more focused analysis and to better understand the association between the keywords, further screening was required.

**Figure 6:**
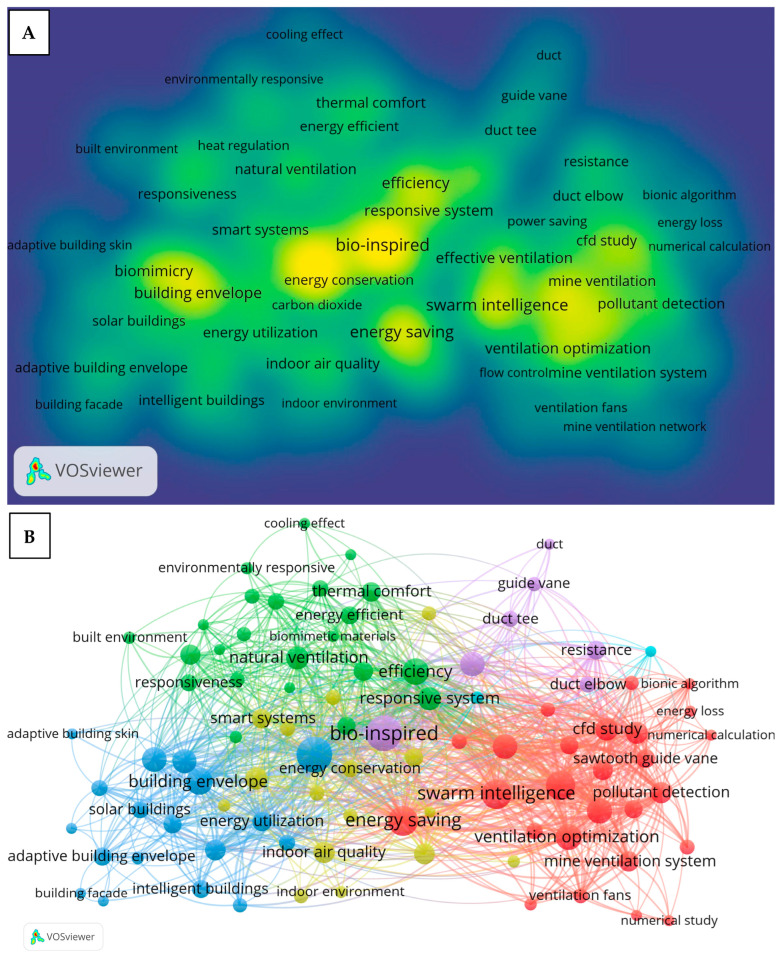
Keyword co-occurrence maps after the screening process: (**A**) density visualization; (**B**) network visualization.

After the screening process, the keywords displayed in the density visualization map were more directly related to the ventilation systems and their components, with a total of 92 terms retained based on the occurrence threshold of 10. As shown in Figure 6A, the most frequently occurring terms included bio-inspired, biomimicry, ventilation optimization, swarm intelligence, energy saving, energy conservation, efficiency, building envelope, and responsive system. Figure 6B shows that these keywords were grouped into five interconnected clusters: the red cluster represents ventilation and optimization; the blue cluster focuses on buildings and their components; the green cluster represents the performance outcomes and environmental adaptability of the ventilation and buildings; the purple cluster focuses on the ventilation components such as duct tees and guide vanes; and the yellow cluster represents various aspects of the indoor environment.

**Figure 7:**
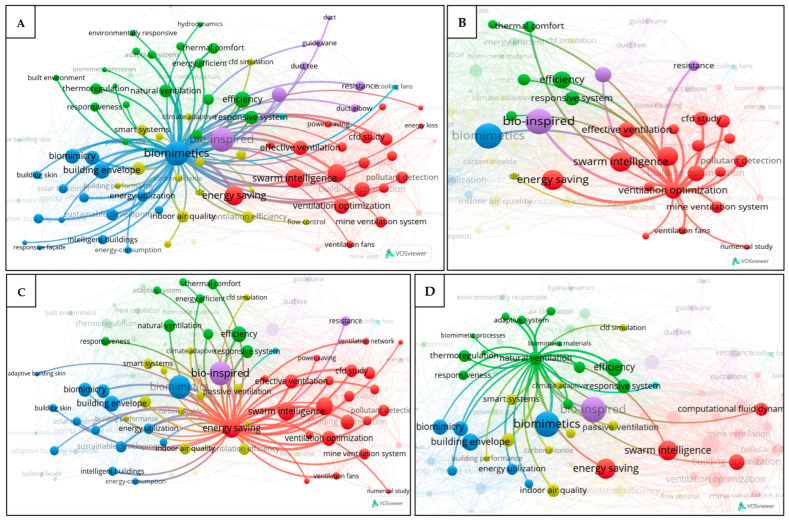
Links and co-occurrence of keywords: (**A**) biomimetics and bio-inspired; (**B**) ventilation optimization; (**C**) energy saving; (**D**) natural ventilation.

**Figure 8:**
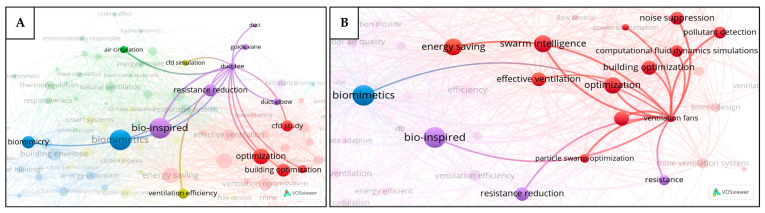
Links and co-occurrence of keywords: (**A**) duct tee, duct, and guide vane; (**B**) ventilation fans.

Figure 7A shows that the keywords “bio-inspired” and “biomimetics” are linked to all clusters, which implies the significance of bio-inspiration across various aspects of the ventilation systems. The keyword “ventilation optimization” is closely associated with ventilation fans, swarm intelligence, mine ventilation systems, effective ventilation, and energy saving, as shown in Figure 7B. This indicates an interest of the research community in optimizing ventilation systems, including mine ventilation, through the application of bio-inspired algorithms such as swarm intelligence, with the goal of improving their effectiveness and energy efficiency. Similarly, the term “energy saving” (Figure 7C) is strongly connected to responsive systems, smart systems, energy-efficient, intelligent buildings, and energy utilization, suggesting that the energy saving strategies in ventilation and building designs are closely tied to the development of smart, intelligent, and responsive systems. At the same time, it also has a strong connection with passive ventilation, suggesting that this approach, which is generally simpler to operate and implement, plays a significant role in achieving energy savings. The keyword “natural ventilation” (Figure 7D) shows strong links to smart systems, responsive systems, and passive ventilation. The keywords “duct tee” and “duct elbow” (Figure 8A) are both associated with the resistance reduction, implying that the research in this area has focused on minimizing the resistance in duct components to improve the ventilation performance. Finally, the keyword “ventilation fans” (Figure 8B) is closely associated with optimization, noise suppression, resistance reduction, biomimetics, and bio-inspired design, implying that the research community aims to improve fan performance through nature-based strategies, such as reducing aerodynamic resistance, suppressing noise, and enhancing the overall efficiency.

#### 2.3.2. Comparative Analysis

**Figure 9:**
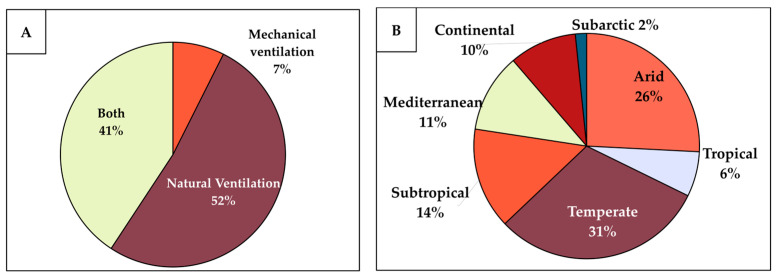
Comparative analysis based on (**A**) ventilation types; (**B**) climate types.

To better understand the progress and applications of the bio-inspired ventilation research, a comparative analysis of the fully screened documents was conducted in this study, focusing on the research methods, structural applications, relevance to mechanical versus natural ventilation, and suitability across various climate conditions. This analysis revealed a clear trend: most bio-inspired ventilation designs are intended for natural ventilation, with fewer being applicable to both natural and mechanical systems, and only a small fraction is designed exclusively for mechanical ventilation, as shown in Figure 9A. This trend is likely due to the inherent compatibility of bio-inspired strategies with natural ventilation. Organisms in nature often utilize simple yet effective strategies of structural or design solutions to harness natural-force-driven ventilation, as is evident in the prairie dog burrows [34], termite mounds [35], and leaf-cutter ants’ nests [36]. Generally, natural ventilation systems require less maintenance and consume significantly less energy than mechanical ventilation systems [37]. The bio-inspired ventilation research is often motivated by the goal of enhancing the energy efficiency in buildings [28,29]. While the bio-inspired principles can be integrated into the mechanical ventilation, their application is inherently challenging since mechanical systems rely on active energy consumption, which is fundamentally different from the passive nature of the biological ventilation strategies. These factors collectively contribute to the higher prevalence of bio-inspired research on natural ventilation compared to mechanical systems.

Most of the studies reviewed were conducted in temperate and arid climate regions (Figure 9B), particularly in China, while very few focus on tropical countries. This trend is largely due to factors such as rapid urbanization [38], government policies promoting energy-efficient buildings [39], and climate diversity in China [40], which likely encourage the research into more adaptive, effective, and energy-efficient ventilation strategies. In contrast, many fully tropical countries, such as those in Asia, continue to rely on mechanical ventilation due to the challenges of high humidity and year-round warm temperatures [41], which make natural ventilation less effective [42]. Additionally, urban heat islands in major tropical cities trap heat [43], further reducing the efficiency of the natural ventilation in providing sufficient thermal comfort [44]. As a result, the bio-inspired ventilation research, which primarily focuses on enhancing natural ventilation, is less prevalent in tropical regions, where the need for mechanical cooling remains dominant.

**Figure 10:**
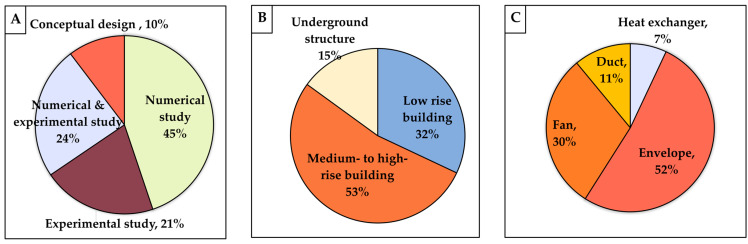
Comparative analysis of the studies according to (**A**) study type; (**B**) built environment type; (**C**) ventilation systems’ component types.

The comparison also showed that nearly half of the studies utilized numerical analysis, with only 21% conducting experimental and 24% implementing combined numerical-experimental approaches, as shown in Figure 10A. This is likely due to the fact that although bio-inspired ventilation strategies offer innovative solutions for enhancing the airflow in buildings, their lack of direct control makes it challenging to test them experimentally or implement them in real-world settings. Computational methods such as CFD simulations are often used to model the airflow behavior before the experimental testing.

In terms of built environment types, the most dominant category incorporating bio-inspired strategies is medium- to high-rise buildings, which is followed by low-rise buildings, as shown in Figure 10B. This trend can be attributed to the greater energy demands associated with ventilating taller buildings. High-rise structures typically require more advanced ventilation strategies due to increased internal heat gains, higher occupant density, and greater reliance on mechanical systems. Moreover, natural ventilation forces, such as pressure gradients and stack-induced convection, are more pronounced in taller buildings. As a building increases in height, the vertical temperature and pressure differentials between floors become more significant, creating stronger driving forces that can be harnessed to enhance passive ventilation systems. In contrast, although underground structures, such as mines or tunnels, often present equally or even more demanding ventilation requirements, the application of bio-inspired ventilation in such environments remains limited. This may be due to the high-risk nature of underground spaces, where implementing novel or unconventional systems requires extensive validation and stricter compliance with safety regulations. Nonetheless, research into the use of bio-inspired or nature-inspired algorithms for optimizing ventilation performance is gradually increasing in underground infrastructure, indicating a growing interest in applying biomimetic principles beyond conventional building contexts.

In terms of ventilation or building component types, the building envelope is the dominant category incorporating bio-inspired strategies, as shown in Figure 10C. This is likely due to their direct connection and interaction with the natural ventilation, as they serve as the primary barrier to the external environment. Bio-inspired envelopes often integrate features such as porous materials, adaptive apertures, or environmentally responsive elements that enhance natural ventilation and thermal regulation. Fans also represent a relatively dominant area of research, as fluid dynamic studies aimed at improving fan efficiency have been ongoing for decades. Many of these investigations draw inspiration from biological forms, such as bird wings, fish fins, and insect flapping mechanisms, to improve aerodynamic performance, reduce noise, and increase energy efficiency in mechanical ventilation systems.

The analysis presented above provided an understanding on how the field has developed and where further work is needed. Overall, the findings showed that bio-inspired ventilation research is strongly oriented towards improving ventilation efficiency, and this is pursued through the optimization of the ventilation system, its components, and the building itself, aiming to reduce the energy consumption, enhance the responsiveness to the environmental changes, enable a smarter operation, and improve the air distribution. These findings raise a fundamental question: Should we shift more decisively towards the bio-inspired solutions in the pursuit of achieving a higher efficiency of the ventilation systems? Nature’s designs and solutions, refined through millions of years of evolution, could provide valuable models for improving a wide range of engineered systems, including those for ventilation.

## 3. Bio-Inspired Passive Ventilation

The passive mechanisms for air regulation or ventilation in the built environment are often more energy-efficient than active ones because they operate with minimal power requirements and do not rely on mechanical components such as fans [45,46,47,48,49]. However, these systems are generally less controllable and more dependent on the external environmental conditions, which can sometimes compromise the occupant comfort [45,50]. Given that passive ventilation strategies have long existed in nature, it is valuable to examine how different organisms achieve self-regulated ventilation through structural adaptations. Accordingly, this section explores bio-inspired passive ventilation and discusses its current applications in the built environment, highlighting the advantages and limitations of each approach.

### 3.1. Ventilation Mechanism

#### 3.1.1. Pressure-Gradient-Based Mechanism

- Velocity-difference-induced pressure gradient

**Figure 11:**
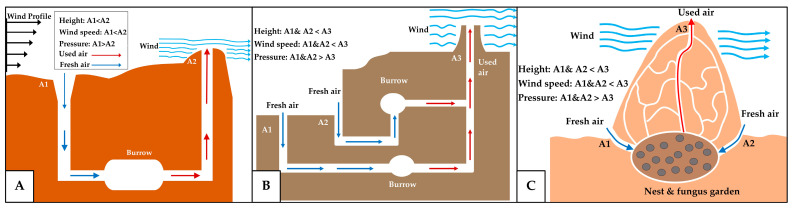
The velocity-difference-induced pressure gradient ventilation mechanism of (**A**) prairie dog burrow; (**B**) crayfish burrow; (**C**) termite mound.

The flow of air driven by pressure gradients is one of the most fundamental physical principles underlying the ventilation mechanism. It occurs when a difference in air pressure exists between two or more regions within an environment, causing the air to move from the higher-pressure zones toward the lower-pressure zones [51]. One of the primary causes of such pressure differences is the variation in the airflow speed between these regions, which creates the corresponding differences in static pressure. This mechanism plays a crucial role in regulating the airflow in many animal dwellings, such as in prairie dog burrows, where the pressure-gradient-driven flow is achieved simply through varying the height and size of the entrance and exit openings [52,53,54]. As shown in Figure 11A, the burrow consists of interconnected passages with one entrance shaped narrowly and located at a higher elevation, while another with a wider opening is positioned lower [34,53,54]. Following the Bernoulli principle, the lower entrance, which is exposed to slower wind speeds, experiences higher pressure, thus drawing air into the burrow, which then exits through the elevated entrance, where the wind speed is higher and the pressure is lower. This system allows for even a light breeze at the lower opening to ventilate the burrow [52,54]. A similar mechanism is observed in the burrows of crayfish, which typically comprise a chimney-like vertical shaft with an upper opening and one or more lower-level entrances (Figure 11B). The chimney, which experiences negative pressure due to being exposed to a higher wind speed, draws the air out of the burrow, while the lower openings allow the fresh air to flow inward [55].

Several studies suggest that a similar principle also operates in termite mounds, although their structural configuration differs slightly. The termite mounds are typically built to considerable heights, often several meters above the ground, making them comparable, on a relative scale, to skyscrapers in human architecture. The mechanisms governing the air circulation and gas exchange within these mounds can vary depending on the geographic location, environmental temperature, and mound architecture [56,57,58]. Nevertheless, the wind-induced pressure gradient (Figure 11C), which arises from the variations in the height and size of the openings, is recognized as one of the primary ventilation mechanisms, particularly in the mounds with open chimneys or those featuring holes and thin porous structures, such as the mounds of *Macrotermes jeanneli*, *M. subhyalinus*, and *Odontotermes transvaalensis* [54,59,60,61,62]. For this mechanism to function effectively and be sustained, at least two openings are required, one positioned at a higher elevation and another positioned lower, to enable a continuous airflow [35,57,63].

b. Boundary-layer-driven pressure gradient

**Figure 12:**
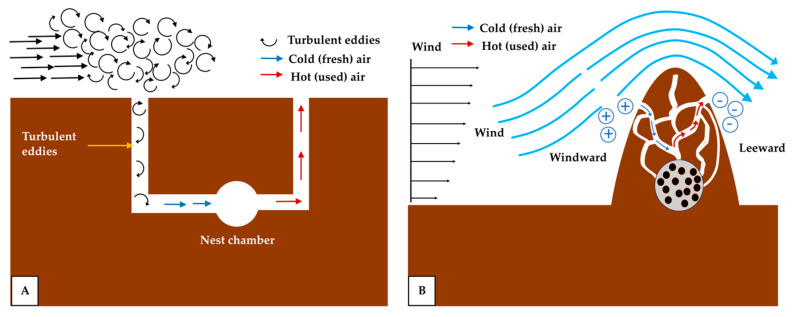
(**A**) Turbulent-eddy-driven ventilation mechanism in Sundevall’s jird burrows; (**B**) boundary-layer-driven pressure gradient in termite mounds, showing that the windward side experiences positive pressure, drawing air into the mound, while the leeward side experiences negative pressure, expelling stale air from the mound.

Another type of pressure-gradient-driven ventilation mechanism has been documented in Sundevall’s jird burrows, where the airflow is governed by the interaction between the burrow entrances and the turbulent eddies, originating from the boundary layer near the ground surface [64]. This mechanism differs from that of prairie dog burrows, which exhibit an unidirectional airflow and require openings at different heights [54,65]. Here, the ventilation mechanism is driven by instantaneous fluctuations in the wind velocity within the turbulent regime, which drive the intermittent air exchange between the burrow and the atmosphere. The turbulent boundary layer contains eddies of varying sizes that detach from the main flow and impinge upon the ground surface. At a large scale, these eddies generate wind gusts as they pass over the burrow entrance, creating pressure differences that drive the pressure-gradient-induced airflow. At a smaller scale, the eddies can penetrate the burrow entrances (Figure 12A), mixing the interior air with the fresh atmospheric air, thereby lowering the CO_2_ concentrations and replenishing the O_2_ levels. However, the eddy penetration does not directly reach the nest chamber; instead, it enters the tunnel corridor, from which the fresh air subsequently flows into the nest chamber [64,65]. In termite mounds, the pressure gradient induced by the atmospheric boundary layer becomes even more pronounced, as the mound extends for several meters above the ground surface. As shown in Figure 12B, the windward side of the mound experiences a higher pressure, while the leeward side is subjected to a lower pressure, generating a continuous circulation of the air through the mound [57,66].

#### 3.1.2. Convection-Based Mechanism

- Metabolic-heat-driven thermal convection

**Figure 13:**
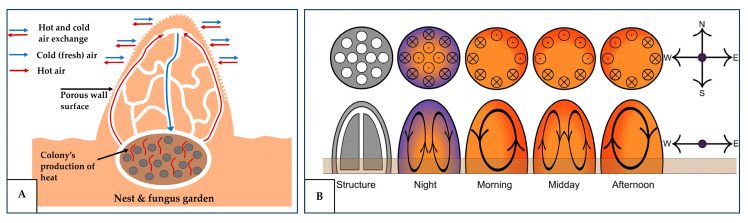
(**A**) Metabolic-heat-driven thermal convection of termite mounds; (**B**) solar-heating-driven thermal convection of termite mounds. Reproduced with permission from [73].

In animal burrows, the rate of oxygen depletion and carbon dioxide accumulation depends largely on how rapidly these gases are consumed or produced. The consumption of oxygen and production of CO_2_ are governed by animals’ metabolic rates, which vary among species according to their body size and activity levels [67,68]. The heat released from animal metabolism contributes to one of the key passive ventilation mechanisms in nature, namely thermal convection, also known as a thermosiphon flow. This mechanism is hypothesized to occur in a variety of species, including termites [69] and the northern flicker [70]. In termite mounds, this mechanism is most prevalent in closed mounds without chimneys, where the air movement is generated by the metabolic heat produced by the colony located in the lower part of the mound [69]. As shown in Figure 13A, the heat warms the surrounding air, making it buoyant and causing it to rise toward the top of the mound, where it mixes with the fresh air entering through the porous upper structure and accumulates additional water vapor. The now denser and cooler air then descends toward the nest, creating a continuous convective circulation that supplies fresh air to the colony [35,69]. In northern flicker nests, the cavity air has been observed to be, on average, 7.1 °C warmer than the outside air [71]. This temperature gradient effectively doubles the rate of the gas exchange between the cavity and the surrounding air, improving the diffusion efficiency and mitigating the risk of the CO_2_ buildup within the nest or mound [67]. However, this thermally induced convection is not always sustainable, as it depends strongly on the external conditions. For instance, when the temperature difference between the interior and exterior of the dwelling is minimal, the resulting convection may be insufficient to meet the ventilation requirements. Also, maintaining a sufficient temperature gradient to drive the airflow can sometimes compromise the occupant comfort.

b. Solar-heating-driven thermal convection

In some animal burrows, in addition to metabolic-heat-driven thermal convection, temperature differences arising from the periodic solar heating or the ground thermal gradients also contribute significantly to the ventilation [56,67,68,72]. The solar heating creates a temperature gradient between the air inside the burrow, the burrow walls, and the surrounding soil surface. For example, in the burrows of Sundevall’s jird (*Meriones crassus*), the temperature of the air and burrow wall is cooler than that of the surrounding soil at night, while the upper portion of the burrow remains cooler than the deeper layers. This pattern reverses during the day, when the burrow air becomes warmer than the soil, and the upper section is warmer than the lower one. These temperature gradients drive the convective airflow within the burrow. Such solar-induced thermal convection becomes more important during calm, windless periods, particularly between sunset and sunrise, as reported in these studies [68].

A similar principle has been observed in some termite species, including *Odontotermes obesus* and *Macrotermes michaelseni* [73,74]. Although these two species inhabit very different environments, with *O. obesus* typically being found in the relatively moderate temperate regions and *M. michaelseni* occurring in the semi-arid areas exposed to stronger winds, both have developed mound architectures that effectively harness the periodic solar heating to drive the convective air exchange [73]. In both mounds, the air circulates as a convective cell that reverses direction following the daily temperature oscillations, as shown in Figure 13B. During the day, the centre of the mound remains cooler than its periphery, causing the warm air along the outer regions to rise while the cooler air descends to the centre of the mound. At night, the pattern reverses, with the cooler periphery and warmer center producing an upward air flow in the center and a downward airflow along the periphery [73,74]. However, this circulation pattern can be further influenced by the asymmetric solar exposure, as the direct sunlight heats different mound surfaces throughout the day. For example, in the morning, the east-facing surface warms more rapidly than the west, potentially slightly altering the flow pattern [73]. In the context of the built environment, these mechanisms can inform the building orientation and design strategies that utilize the diurnal temperature variations and solar exposure to promote a passive air circulation.

#### 3.1.3. Combined Pressure-Driven and Convection-Driven Ventilation Mechanisms

**Figure 14:**
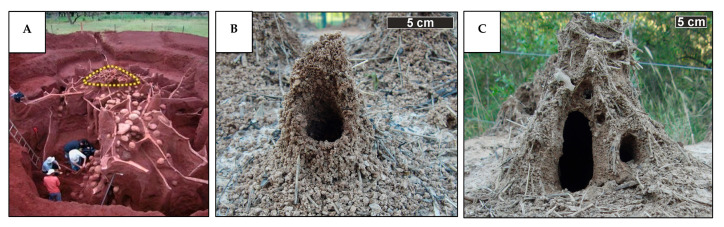
(**A**) Leaf-cutter ants’ nests. Reproduced with permission from [78]; (**B**) ant nest with only one turret opening; (**C**) ant nest with a turret and multiple openings. (**B**,**C**) Reproduced from [80], CC BY 4.0.

In nature, many ventilation mechanisms rarely function as isolated systems; rather, they often operate through the integration of multiple mechanisms. For instance, in the nests of leaf-cutting ants, two distinct ventilation principles coexist. The dominant mechanism is pressure-gradient-driven ventilation, induced by differences in wind speed across the nest surface [36]. Ant colonies construct some of the largest and most complex underground nests (Figure 14A), which can house up to 5 million individuals [75,76,77]. These nests rely on passive ventilation to maintain optimal conditions for fungus cultivation, including temperatures of 25–30 °C, suitable humidity levels, and efficient oxygen exchange [78,79,80,81,82]. The nest is equipped with turrets with single or multiple openings, as shown in Figure 14B,C. Ventilation occurs through a top-mounted turret, which exhausts CO_2_-rich air, while lower openings draw in cooler, oxygen-rich air [36,76,83].

In addition to the wind-driven mechanism, several studies suggest a secondary contribution from thermally induced convection, in which temperature gradients between the nest interior and the surrounding soil generate buoyancy forces that drive upward airflow [75,84]. However, this thermal component is considered minimal, as leaf-cutting ants are highly sensitive to elevated temperatures, which can be detrimental to both the colony and their cultivated fungus [82,85].

#### 3.1.4. Diffusion-Based Mechanism

Fluid molecules are in constant random motion, continuously colliding with one another and changing direction while conserving momentum. This random molecular motion results in the movement of atoms, molecules, or small particles from regions of higher concentration to regions of lower concentration. This process is commonly referred to as diffusion [86,87]. In the absence of convection, diffusion serves as the primary means of molecular transport over short distances, although it becomes inefficient for larger-scale transport due to its slow rate [87]. Diffusion plays a vital role in many organisms, which enables not only gas exchange but also the transport of solutes and nutrients.

In humans and other mammals, diffusive gas exchange takes place in the alveoli of the lungs, where oxygen diffuses from the inhaled air into the deoxygenated blood, while carbon dioxide moves in the opposite direction [88,89,90]. Diffusion also occurs in the kidney, where processes such as urea and water reabsorption occur passively across selectively permeable membranes, particularly within the Loop of Henle [91]. This U-shaped structure, located in the kidney’s medulla, consists of a thin descending limb, a thin ascending limb, and a thick ascending limb that work together to form a counter-current system [92,93]. This system establishes a concentration gradient in the surrounding tissue, which enables efficient water reabsorption with minimal energy use [91].

In plants, diffusion occurs through the stomata, where carbon dioxide enters and water vapor exits during photosynthesis [94,95,96]. These tiny pores on the leaf surface regulate gas exchange by controlling their size, number, and distribution in response to environmental signals such as light intensity, atmospheric CO_2_ concentration, and plant hormones [94]. This regulation allows plants to balance CO_2_ uptake for photosynthesis with water loss through evaporation. For example, when the concentration of CO_2_ in the atmosphere is low, the plants increase the density of stomata but at the same time decrease their size. Conversely, when CO_2_ levels are high, fewer stomata are needed to meet photosynthetic demand, so stomatal density generally decreases [97,98]. Although diffusion alone is less effective as a ventilation mechanism for built environments due to its limited transport rate, it remains valuable in specific applications, such as for heat and mass exchange. For example, diffusion-based mechanisms can be applied in heat recovery ventilation systems, where thermal energy from exhaust air is transferred to incoming fresh air, thereby reducing overall energy consumption during colder seasons.

### 3.2. Application of Bio-Inspired Passive Ventilation Mechanisms to the Built Environment

The bio-inspired passive ventilation mechanisms have been proposed across a wide range of applications, from macroscale applications, such as buildings and urban layouts, to microscale applications, such as in the building envelope and its components. They have also been explored in both outdoor and indoor environments, as well as in underground spaces, where they are primarily intended to enhance natural ventilation performance and consequently contribute to reducing energy consumption.

#### 3.2.1. Macroscale Application

- Buildings

**Figure 15:**
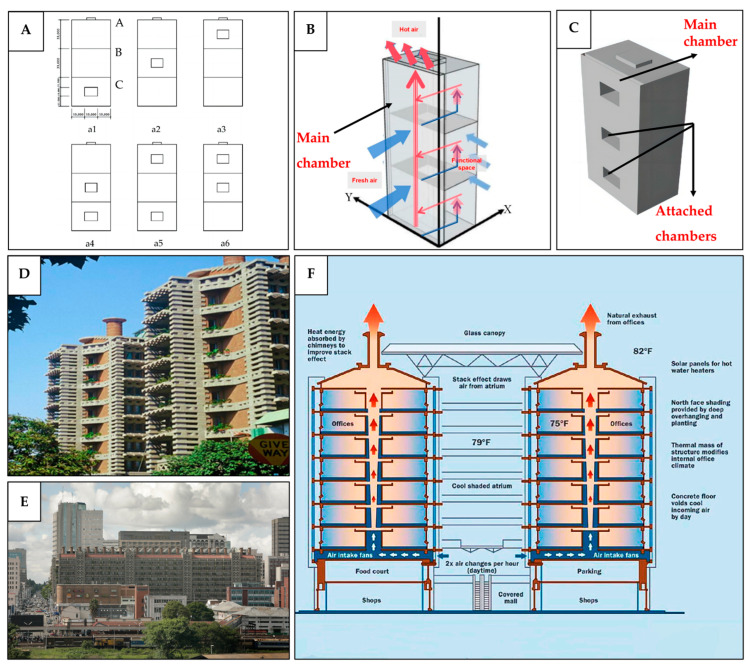
(**A**) Six termite-mound-inspired ventilation arrangements featuring different numbers of chambers at various building levels; (**B**) schematic depiction of the ventilation mechanism of termite-mound-inspired chambers in a high-rise building; (**C**) main chamber and single attached chamber of termite-mound-inspired ventilation. (**A**–**C**) Reproduced from [99], CC BY 4.0. (**D**) Close view of the Eastgate Centre showing the incorporation of a porous façade element into the building. Reproduced from [103], CC BY 4.0. (**E**) Distant view of the Eastgate Centre showing roof-mounted chimneys at the top, which are open to the outside air. Reproduced from [104], CC BY-SA 3.0; (**F**) Ventilation mechanism of the Eastgate Centre. Reproduced with permission from [105].

In the context of buildings, bio-inspired passive ventilation mechanisms have primarily been proposed for medium- to high-rise structures. This trend is likely due to the energy efficiency being more critical in taller buildings, where mechanical ventilation typically accounts for a larger share of total energy use. In addition, many bio-inspired passive systems are based on pressure-gradient-based principles, which are inherently more effective in structures with greater height differences. Taller buildings are exposed to stronger wind-speed variations than those experienced by low-rise buildings, making pressure-gradient-induced airflow more pronounced and effective. For example, this approach has been explored through designs that incorporate chambers of varying numbers at different building levels (Figure 15A) to create a pressure difference and hence improve natural ventilation efficiency [99]. The chambers are connected to the functional spaces through the ventilation openings that draw the stale air into the main chambers, from which it is discharged with the help of the wind-driven pressure difference (Figure 15B). The simulation for China’s climate condition showed that the models featuring a main chamber combined with double-attached chambers, as shown in Figure 15C, were the most effective, achieving a maximum wind speed difference of 0.19 m/s between the floors and providing a stable wind environment. Even simpler configurations, such as the main chamber alone or the main chamber with a single attached chamber, still generated higher airflow rates within the interior space compared to an original chamber-free high-rise building [99].

The combined principles of thermosiphon and pressure-gradient-induced flow have also been proposed. A prominent example is the Eastgate Centre in Harare, Zimbabwe (Figure 15D,E), designed by Mick Pearce, which integrates both mechanisms to minimize dependence on conventional air-conditioning systems. The buildings are constructed from concrete slabs and bricks that can absorb heat without significant changes in their temperature due to their high thermal mass. Integrated into the design are tall chimneys, which are open to the fresh air at the top, and low-power fans that assist in drawing in the outside air [100,101]. At night, these fans pull the cool air indoors and distribute it through the hollow floors, allowing the concrete to absorb the coolness and reduce the temperature of the circulating air. During the day, the heat from the occupants, equipment, and the surrounding environment warms the indoor air, triggering a thermosiphon effect that moves the warm air through the ceilings and upward into the chimneys, from which it is expelled outside [63]. This design is estimated to use only 10% of the energy consumed by buildings of a similar size in the region [102]. Figure 15F illustrates the termite mounds-inspired ventilation mechanism of the Eastgate Centre.

In addition to indoor applications, this principle has also been proposed to enhance the outdoor thermal comfort of residential blocks in New Aswan, Egypt, through strategic re-arrangement and re-orientation of the buildings. It was found that aligning the buildings along the east–west axis created pressure differences that improved the natural ventilation, thus reducing the Universal Thermal Climate Index (UTCI) and saving approximately 10,407.29 kWh of energy during the summer months [106]. However, this model is better suited for building blocks with planned gaps between them to allow for pressure differences to develop. If buildings are constructed as a single attached block without gaps, the intended ventilation effect may be largely lost. In addition, since this model was developed for hot and arid climate conditions, its applicability to cold regions, where the goal is to reduce the heating loads, could be less effective.

**Figure 16:**
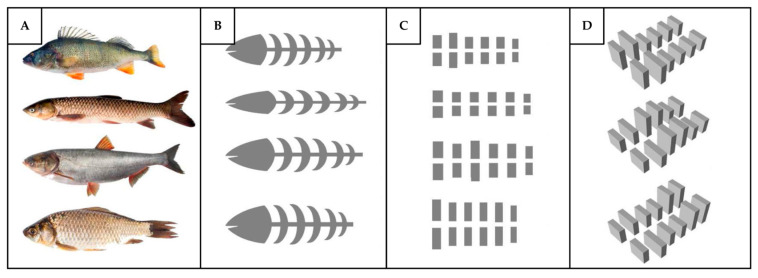
Schematic diagram of (**A**) different forms of fish; (**B**) fishbone shape; (**C**) building layout inspired by the fishbone shape; (**D**) building height inspired by the fishbone shape. (**A**–**D**) Reproduced with permission from [107].

Likewise, in a study by Wei et al. [107], the pressure gradient principle was applied to improve outdoor ventilation. The authors developed a fishbone-inspired bionic architectural layout designed to enhance natural ventilation efficiency in high-density residential communities. The ventilation mechanism primarily relies on wind-induced pressure gradient, where strategic variations in building height and spacing enhance the flow of air through the ventilation corridors, similar to fishbone branches. Using computational fluid dynamics (CFD), nine design configurations were simulated, varying in both layout (single- and double-row fishbone forms) and building height. The results showed that the bio-inspired building layout and height significantly improved the pedestrian wind conditions, reduced the static air zones, enhanced the natural ventilation, and decreased the wind pressure on the windward buildings by 20%. To further optimize the wind environment, the study recommended placing the longer buildings at the center and positioning the taller buildings farther from the prevailing wind direction. While the results are promising, this study did not account for the key parameters of ventilation comfort, such as the temperature, draft, and age of the air, which play an important role in assessing the performance of ventilation systems. In particular, although wind can enhance the heat transfer between the human body and the surrounding environment, it may also increase the draft sensation. As a result, occupants in windy environments often experience a lower thermal comfort compared to those in windless conditions [108], even when the overall cooling effect is beneficial. Future research should therefore examine how the fishbone building layouts influence such human comfort indices to better understand the practical implications of this bio-inspired ventilation strategy. Figure 16 shows the schematic diagram of different fishbone geometries and their translation into building block layouts and heights.

In a more recent study, Ramasam et al. [109] applied the same strategies for a multi-story residential building in Chennai, India. By incorporating ventilation pipes at both the bottom and top of the structure, a stack effect was generated that enhanced the air circulation and reduced the indoor temperatures by up to 30%, maintaining a stable range of 28–32 °C even when the outdoor temperatures reached 40 °C.

b. Underground structure

Bio-inspired passive ventilation mechanisms have also been studied for underground structures, such as tunnels [52] and underground dwellings [110]. In the context of tunnels, the strategy has been proposed for subway stations, where numerical studies based on fluid dynamics laws were conducted to assess the effectiveness of passive ventilation in improving the air change rate. The results showed that elevating one tunnel opening, along with varying the heights, shapes, and sizes of the other openings, generates a pressure difference that drives the natural ventilation, thereby increasing both the air change rate (ACH) and the air flow rate in the tunnels [52]. However, the assumptions and simplifications applied in this study might not fully capture the complex and interconnected nature of the real-world tunnel networks, thus reducing the effectiveness of the proposed design in practical applications. For further research, laboratory testing in conjunction with computational fluid dynamics (CFD) analysis is recommended to validate the model and improve its reliability.

**Figure 17:**
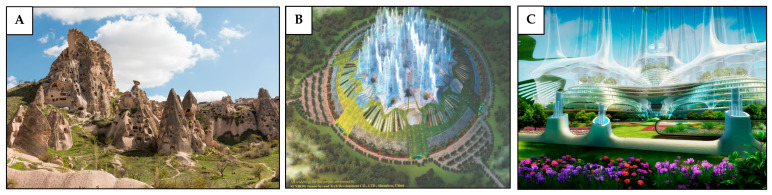
(**A**) The structure in Cappadocia that resembles an ant nest. Reproduced from [113], CC BY-SA 4.0. (**B**,**C**) The design concept of a global health center inspired by ant nests. (**B**,**C**) Reproduced from [112], CC BY 4.0.

Many human-made structures, particularly those built underground, resemble the architecture and ventilation systems found in nature, such as leaf-cutting ant nests, even if not explicitly designed with this inspiration in mind. A remarkable example of this is the ancient underground city in Cappadocia, Turkey, which features interconnected conduits, ventilation shafts and chimneys that open to the fresh air (Figure 17A) for natural air circulation, allowing the city to remain well-ventilated with minimal need for an auxiliary air supply [110]. Another example is the Global Health Center concept (Figure 17B,C), proposed by the State Key Laboratory at the South China University of Technology. The structure spans approximately 120 m in depth and 500 m in diameter, designed to accommodate up to 2000 people. Its ventilation strategy employs concentric cooling walls that act as cool heat sinks that cool the incoming air, while a central chimney expels the heat and stale air to maintain a stable indoor temperature between 18 °C and 22 °C [111,112]. While the ant nests offer valuable insights for the passive underground ventilation, applying these principles to deep underground dwellings pose serious challenges. The geological variability, flooding risks, and gas accumulation require complex mitigation systems that reduce the practicality of such designs for permanent habitation [110]. Therefore, bio-inspired ventilation might be more suitable for underground tunnels, mines or workspaces, where people stay temporarily and where improved airflow can improve the energy efficiency.

#### 3.2.2. Microscale Application

At the microscale or component level, research has primarily focused on the application of passive ventilation mechanisms within the building envelope, particularly façades and walls. For example, Paar and Petutschnigg [34] developed a bio-inspired ventilated façade with extruded upper and lower openings to cool buildings and mitigate the urban heat island effect. The initial calculations and laboratory tests on reduced-scale models, based on summer temperature conditions in central European cities, demonstrated that the bio-inspired ventilated façade design nearly doubled the airflow speed within the ventilated slot compared to the conventional models. As a result, wall surface temperatures decreased significantly, reducing the building’s cooling load and improving the building’s energy savings. However, because the design relies on the wind, its effectiveness across different climates remains uncertain, as the wind conditions vary considerably between regions. Moreover, structural limitations and scaling challenges may arise when applied to larger buildings, where ensuring a consistent performance under more complex conditions would become increasingly difficult.

Such passive ventilation mechanisms have also been proposed for application in the hot desert climate [114]. To mimic the elevated entrance of prairie dog burrows, an opening called the “top mound opening” was placed at the top of the double skin façade, rather than placing it on the side. This configuration aimed to reduce the inner skin surface temperature of buildings by accelerating the airflow within the ventilated slot. The simulation results indicated that the bio-inspired design increased the air velocity within the slot by approximately 350% compared to the baseline design, while also lowering the inner skin surface temperature by 5.4 °C under full sun conditions. However, the study was limited to façades in low-rise buildings, and therefore, the applicability of this design for other types of buildings, such as high-rise and mid-rise, is uncertain.

In addition to the pressure-gradient-based mechanism, the diffusion-based mechanism has also been explored for its application at the component level. Lee and Lee [115] studied an optimum opening configuration of pneumatic façade to enhance the natural ventilation into a built environment, ensure desirable airflow diffusivity and improve occupant comfort. The results showed that, to generate indoor airflow closer to the recommended levels for occupant comfort, configurations with larger openings should be used under the lower wind speeds, while smaller openings are more suitable at higher wind speeds. In addition, the larger openings should be positioned toward the windward side, with the smaller openings placed on the leeward side. However, it is important to note that this study relied solely on CFD simulations. Since different turbulence models can produce varying results, a more detailed exploration and discussion of the turbulence model selection would strengthen the validity of the findings. Beyond the terrestrial applications, the concept of adaptive façades could also be extended to space habitats, where not only the external envelope but also the internal partitions could dynamically adjust to optimize the ventilation according to the occupants’ needs.

**Figure 18:**
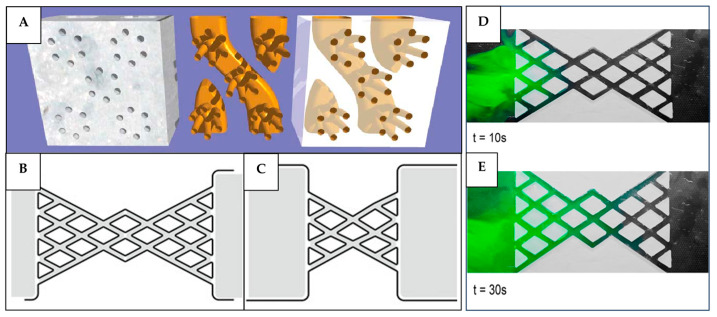
(**A**) Artificial surface conduits or reticulated tunnels. Reproduced from [63], CC BY-NC-ND 2.5. (**B**) Wide reticulated tunnels. (**C**) Narrow reticulated tunnels. (**D**,**E**) Flow mixing of wide reticulated tunnels at 10 s and 30 s, respectively, showing the mass transport and flow pattern. (**B**–**E**) Reproduced from [116], © 2023 Andréen and Soar, CC BY 4.0.

A more innovative approach involves artificial surface conduits or reticulated tunnels (Figure 18A) integrated into façade systems to create porous walls that facilitate wind-driven natural ventilation [63]. The mechanical actuation through small oscillations can also complement the wind pressure to drive the heat, air, and moisture exchange between a building’s interior and exterior. Andréen and Soar [116] found that reticulated tunnels, both narrow and wide (Figure 18B,C), exhibited a faster flow mixing under high-amplitude oscillations compared to non-reticulated channels. Specific combinations of amplitude and frequency contribute to the turbulence generation, which drives mass transport (Figure 18D,E). The mass transfer was found to be the strongest at oscillation frequencies of 30–40 Hz. However, such reticulated tunnels may be difficult to manufacture precisely to balance the occupant needs or wind conditions. The limited control over the barrier between the interior and exterior of the building may cause drafts and discomfort if the system is fully passive. On the other hand, active oscillation-driven façade systems offer greater control but introduce new challenges: they can be complex and energy-intensive in large-scale applications, as maintaining controlled mechanical vibrations at specific amplitudes and frequencies may offset the energy savings achieved through natural ventilation. Furthermore, the structural durability and acoustic effects of continuous oscillations require further investigation before such systems can be widely implemented.

**Figure 19:**
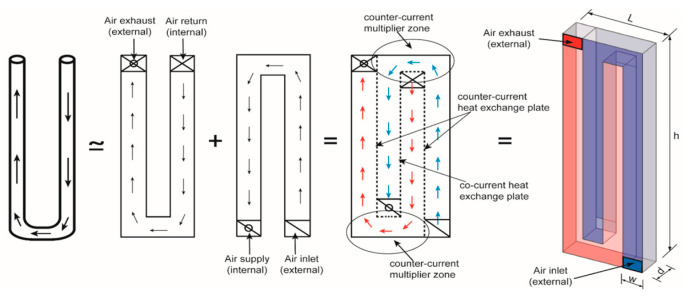
Schematic evolution of the biomimetic heat recovery system, showing the transition from a U-shaped duct with an additional ∩-shaped duct to a merged configuration forming the LoH chamber. Reproduced from [120], CC BY 4.0.

The passive mechanism at the micro-scale has also been applied to enhance heat recovery in natural ventilation. Recovering the waste heat from the ventilation systems can increase the energy efficiency and reduce the cooling and heating demands [117,118,119]. Adamu and Price [120] proposed a biomimetic heat recovery system (Figure 19), inspired by the kidney’s Loop of Henle, which efficiently extracts useful substances before waste elimination. This passive system was integrated into the building envelope to preheat the supply air using indoor heat sources such as occupants, equipment, and lighting. It consisted of a U-shaped duct for the air return and external exhaust, an ∩-shaped duct for the air intake and internal supply, and a Z-shaped aluminum plate at their intersection serving as the heat exchanger. The results showed that with the active heating in place, this bio-inspired heat recovery ventilation reduced the heating energy consumption by 65.7–72.1% compared to the traditional window-ventilated room without a heat recovery system. In the month of January, the system achieved a rate of 0.92 air changes per hour (ACH) and maintained indoor temperatures between 19.3 °C and 22.3 °C. However, the simulation domain was restricted to a single room, whereas the real buildings typically comprise multiple rooms or segmented areas, which could influence the efficiency of the heat recovery systems. It also overlooked the impact of the door opening and closing, a factor that contributes to the unintended cooling or heat losses.

### 3.3. Comparative Summary of the Advantages and Limitations of Bio-Inspired Passive Ventilation Across Different Applications

Bio-inspired passive ventilation mechanisms offer clear benefits but also exhibit context-specific limitations that vary according to the type of structure, implementation strategy, and underlying mechanism employed. For instance, at the Eastgate Centre, the integration of multiple bio-inspired strategies reduced reliance on conventional air conditioning; however, fans were still required to draw in fresh air and flush out hot air at varying intensities during the day and night. Likewise, the use of wind to generate pressure differences has its own limitations, as wind is an inherently variable force, such that its speed and direction fluctuate with time and location. Similarly, thermal convection-driven flow depends on the magnitude and persistence of temperature gradients, which can be difficult to sustain or control in dynamic urban or indoor environments. Table 3 summarizes the advantages and limitations of the key bio-inspired passive mechanisms.

## 4. Bio-Inspired Active Ventilation

### 4.1. Ventilation Mechanism

#### 4.1.1. Forced Convection Induced by Piston-like Movement

In some animal burrows or nests, behavioral adaptations of the inhabitants can actively induce air movement, which contributes to the internal air circulation. For instance, the piston-like movement of animals along the length of their nests can generate a form of forced convection, which promotes the mixing of fresh air entering the nest with the internal air and thereby reduces the accumulation of carbon dioxide [122,123]. This mechanism has been observed in woodpecker nests, where the birds move up and down within their cavities at night at irregular time intervals. It is hypothesized that during these vertical motions, the woodpecker functions as a piston that ventilates the cavity [122]. However, this mechanism might not be as effective during the breeding season or when external wind is absent. The higher number of occupants increases oxygen consumption, and hence hypoxic conditions may develop if this mechanism is not coupled with other ventilation mechanisms. To date, this bio-inspired ventilation mechanism has not been applied or proposed for use in built environments. This may be due to uncertainties regarding its effectiveness in meeting human ventilation requirements, as relying solely on such a mechanism may not provide sufficient air movement. Nevertheless, it holds considerable potential. For example, in environments with adequate wind, a piston-like structure could be designed to generate forced convection, either drawing fresh air into the space from outside or enhancing the mixing of indoor air.

#### 4.1.2. Wing Fanning Induced Forced Airflow

Many social insects, including honey bees, bumblebees, paper wasps, and hornets, often inhabit densely populated cavities where passive ventilation is limited. To maintain suitable temperatures and gas concentrations, these insects have developed behavioral adaptations that actively induce airflow within their nests or hives [124,125,126,127,128,129,130]. In honey bee colonies, when nest temperature or CO_2_ levels exceed certain thresholds, workers gather near the entrance and flap or fan their wings to drive air into and out of the nest, thereby lowering internal temperatures and diluting CO_2_ [124,125,128,130]. In hives with a single aperture, entrance fanning enhances ventilation primarily through the generation of turbulence. This is achieved by fanning inside the hive alongside parallel chains fanning in the opposite directions at the entrance [125]. In nests with large entrances, bees self-organize into groups that separate continuous inflow and outflow regions [130]. They also position themselves strategically according to daily temperature conditions. For example, around midday, when ambient temperature and the nest’s temperature are almost the same, fanning typically breaks into multiple clusters, whereas during cooler periods, a single dominant cluster usually forms [130]. This pattern indicates that solar heating also affects the active ventilation mechanism in some animals. Kinematic studies show that although ventilatory fanning involves wing movements similar to hovering flight, the mechanisms of these two behaviors are different. During ventilatory fanning, the bees flap their wings at a significantly lower frequency and a higher stroke amplitude than in hovering. In this context, the wings act more like impellers than propellers. In this process, air is drawn from above and in front of the bee’s head and redirected along the surface to which the bee clings, forming concentrated jets. When performed collectively, these jets combine into larger streams that can transport air over long distances, thereby improving ventilation [124]. In some cases, bees fan head-to-head in opposite directions to accelerate the dilution of CO_2_ and other gases [125].

#### 4.1.3. Volume Variation Induced Pressure Gradient

Like ventilation systems, the respiratory systems of humans and animals play a crucial role in regulating the gas exchange between the internal and external environments. Understanding these natural systems can therefore provide valuable insights for designing more efficient ventilation in the built environment. Mammalian lungs achieve efficient ventilation by continuously replacing the used air with fresh air from the external environment through the periodic movements of a muscle (the diaphragm) that separates the chest cavity from the abdominal cavity [131,132,133]. During inhalation, the diaphragm contracts and moves downward into the abdominal cavity, increasing the volume of the lungs and thereby reducing the internal air pressure inside the lung to below the atmospheric pressure. As a result, air flows into the lungs from the external environment [132,133,134,135]. During exhalation, the diaphragm relaxes and moves upward to its dome-shaped position, decreasing the lung volume and increasing the internal pressure, which forces air out of the lungs [133,135,136].

A similar volume variation occurs in the heart. With each heartbeat, the heart undergoes rhythmic changes in internal volume that generate the pressure differences responsible for driving blood flow [137,138,139]. When the heart muscle relaxes, the internal chambers expand, causing a drop in internal pressure. This pressure reduction allows fluid to move from the upper chambers into the lower ones, following the natural pressure gradient from higher to lower pressure. As the cycle progresses, the heart muscle contracts, reducing the internal volume of the chambers. This compression increases the internal pressure, forcing one set of valves to close and another set to open, thereby directing the fluid forward into the main arteries [139,140,141,142]. The process resembles a reciprocating pump, where volume reduction during contraction generates sufficient pressure to eject fluid into the circulation system.

A comparable fluid exchange mechanism is observed in simple aquatic organisms such as sea sponges. These organisms lack organs, nervous systems, and muscles and instead rely on an internal canal system composed of chambers that pump water in and out of their bodies, supplying oxygen, nutrients, and food while removing waste products [143]. This pumping process is driven by the flagellated chambers that beat to increase the surface area and facilitate the water flow [144].

### 4.2. Application of Bio-Inspired Active Ventilation Mechanisms to the Built Environment

**Figure 20:**
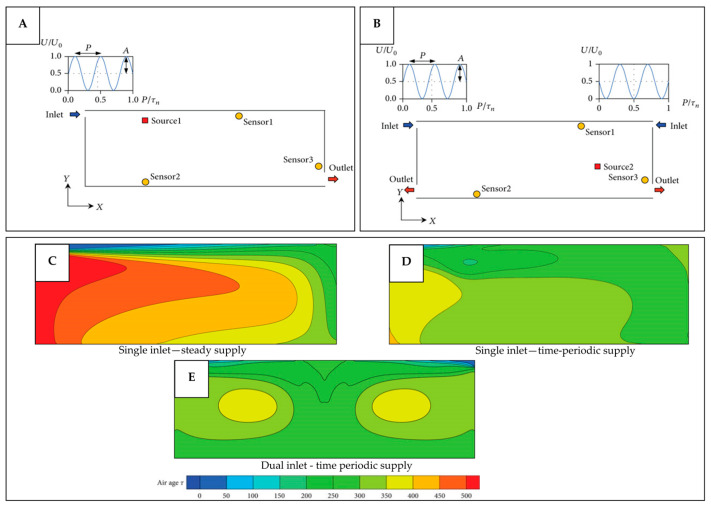
(**A**) Single-inlet time-periodic ventilation model; (**B**) dual-inlet time-periodic ventilation model; (**C**) air-age contour of the steady (constant) supply ventilation; (**D**) air-age contour of the single-inlet time-periodic ventilation; (**E**) air-age contour of the dual-inlet time-periodic ventilation. (**A**–**E**) Reproduced from [145], CC BY 4.0.

Bio-inspired active ventilation systems have received significantly less attention compared to their passive counterparts. This limited exploration may be attributed to the greater complexity involved in replicating active mechanisms found in nature. In biological systems, active ventilation typically relies on coordinated movements, structural flexibility, or muscular actuation to drive fluid flow, which are processes that require energy input and precise control. Translating these dynamic behaviors into engineered systems presents substantial design challenges, particularly in ensuring that the benefits in ventilation performance justify the added mechanical, energy, and maintenance costs. Moreover, achieving the same level of adaptability and responsiveness as observed in systems in nature often demands advanced materials and control strategies, which may not yet be practical or cost-effective for large-scale applications. Nevertheless, several studies have begun to explore bio-inspired active ventilation applications. For example, Zhang et al. [145] proposed a bionic ventilation system inspired by the periodic inhalation and exhalation process in the respiratory systems of humans and animals. Unlike traditional ventilation systems that provide a constant air supply, this bionic system introduced a time-periodic air supply using either a single- or dual-inlet system (Figure 20A,B) regulated by sine or rectangular wave functions. The results showed that both models improve the velocity distribution uniformity, reduce the pollutant concentrations in the stagnant zones, and achieve a higher ventilation efficiency compared to the conventional constant air supply systems. Under both single- and dual-inlet systems, the age of the air of the bionic ventilation was lower than in the traditional constant ventilation, as shown in Figure 20C–E. Among all the models tested, the single-sided supply under the rectangular wave functions demonstrated the best performance in terms of the age of the air and the inhomogeneity coefficient (reduced by 77% compared to a constant supply).

**Figure 21:**
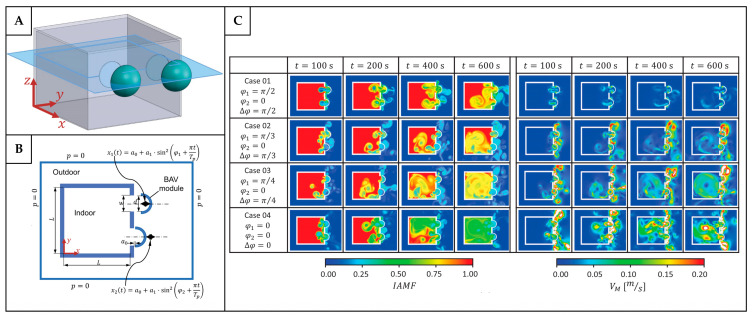
(**A**,**B**) 3D and 2D room model with hemispherical BAV modules, respectively; (**C**) comparison of indoor air mass fraction (IAMF) index and velocity magnitude (V_M_) for different phase shifts (∆φ). (**A**–**C**) Reproduced with permission from [131].

A slightly more advanced example was explored by Marom et al. [131], who developed and numerically studied a biomimetic active ventilation (BAV) for indoor spaces, inspired by human breathing mechanisms, which actively replace the internal air with fresh external air. The CFD domain comprised an unoccupied room with two window openings on one wall and BAV modules translating perpendicular to or rotating about the window openings, as shown in Figure 21A,B. The 2D simulations showed that the dominant parameter influencing the efficiency of the BAV concept was the phase shift between the translating modules. The best performance was registered at the zero-phase shift, where nearly all the indoor air was refreshed with at least 10% outdoor air within about 10 minutes. As shown in Figure 21C, the indoor air mass fraction (IAMF) index, which represents the ratio of the indoor (used) air mass to the total air mass, was the lowest at the zero-phase shift (case 04), while the average indoor velocity magnitude was the highest. This is likely because when the modules move fully in sync, larger volumes of air are exchanged during each cycle. With respect to the geometry, the flat modules were the most effective, as they trap less air within their structure and therefore transport greater air volumes in and out of the room. The 3D simulations further confirmed these findings: although the air movement and dispersion were more complex in three dimensions, the flat BAV modules refreshed the entire room height with at least 10% ambient air in just 10 min. However, this study employed a simplified room geometry and did not account for human occupants, whose presence and exhaled CO_2_ can significantly influence the air quality and ventilation demands. It also overlooked the thermal and acoustic comfort, as the periodic movement of the BAV may cause drafts and noise. The energy requirements were not evaluated; if the energy consumption exceeds that of the conventional mechanical ventilation, its efficiency becomes questionable. Finally, the findings are based solely on CFD simulations without experimental validation, which introduces a high degree of uncertainty.

**Figure 22:**
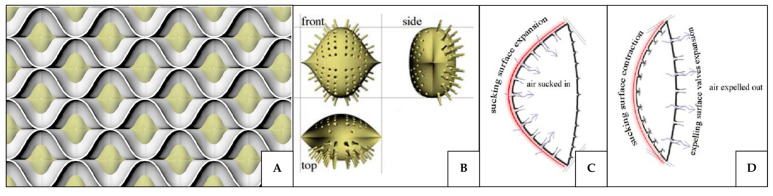
(**A**) Bio-inspired façade with lung-like chambers (LLC); (**B**) front, side, and top views of the lung-like chambers (LLC); (**C**) illustration of lung-like chambers (LLC) during expansion, drawing air in; (**D**) illustration of lung-like chambers (LLC) during contraction, expelling air out. (**A**–**D**) Reproduced with permission from [144].

Inspired by the deep-sea sponges and the respiratory systems of these living organisms, Badarnah and Knaack [144,146] proposed a bio-inspired ventilating envelope with lung-like chambers (LLCs) (Figure 22A,B) that expand and contract to perform air inhalation and exhalation. These LLCs can be made of flexible or elastic materials, allowing them to dynamically adjust their surface area. Sensors can be installed on the inner side of the skin to send signals to the breathing units, enabling them to execute the inhaling and exhaling processes. Each LLC consisted of two surfaces, namely the “sucking” and the “expelling” surfaces, which were interconnected within the basic component. Piezoelectric wires were attached to the sucking surfaces, causing them to expand and create low-pressure zones when electricity was applied. As a result, air entered the breathing chambers through the small holes on the sucking surface (Figure 22C). When the voltage was stopped, the chamber contracted, reducing the volume and creating a high-pressure zone. As a result, air was expelled through the holes on the other sides (Figure 22D). The holes were equipped with the unidirectional valves that ensure the air flows in one direction. When the air was pushed from the inner part of the chamber outward, the valves contracted and closed to prevent the backflow.

To optimize the design, Badarnah, Kadri and Knaack [146] conducted numerical simulations of the breathing chambers by positioning the air expelling (outlet) and air intaking (inlet) units at various locations and using different quantities of units. They found that increasing the number of breathing units does not necessarily improve the uniformity of the air distribution. To achieve better air distribution, it is crucial to position the inlet and outlet at an optimal distance from each other. Additionally, determining the optimal number of components is essential. When the outlet and inlet are placed too close together, the increase in the number of components leads to the fresh air being expelled before proper mixing occurs, hence reducing the overall efficiency.

### 4.3. Comparative Summary of the Advantages and Limitations of Active Ventilation Across Different Applications

Similarly to passive mechanisms, the application and integration of active bio-inspired ventilation systems into the built environment present several limitations and challenges, along with certain advantages. Table 4 summarizes these aspects. For mechanisms that have not yet been proposed or investigated for built environment applications, the advantages and challenges listed represent the anticipated considerations.

## 5. Bio-Inspired Design and Strategies for the Optimization of Ventilation Systems

To integrate nature designs and strategies into ventilation systems for performance optimization, approaches can be broadly classified into component-level and system-level optimization. Component-level optimization focuses on improving individual elements such as fans, ducts, and façades to enhance their aerodynamic, structural, and energy-efficiency characteristics. In contrast, system-level optimization addresses the overall performance of the ventilation network by incorporating control algorithms to achieve higher efficiency and effectiveness.

### 5.1. Component Optimization

#### 5.1.1. Duct

**Figure 23:**
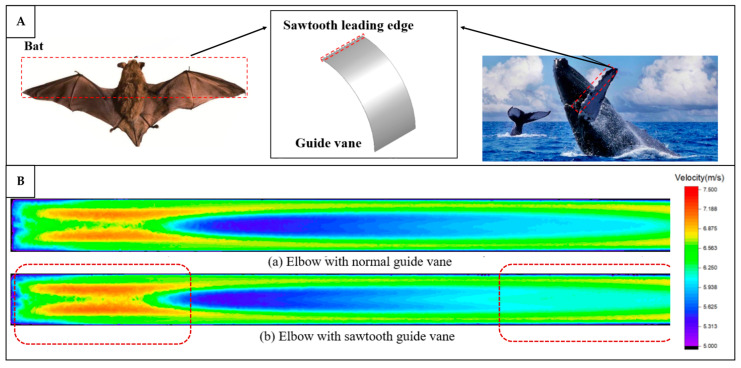
(**A**) Leading edge of duct guide vanes inspired by bat wings and whale pectoral fins; (**B**) comparison of velocity distribution downstream of the duct elbow between normal and biomimetic guide vanes. (**A**,**B**) Reproduced with permission from [148].

A significant source of resistance in ventilation and air-conditioning systems is duct elbows, which contribute significantly to the pressure loss and the increase in the building energy consumption [147,148]. Over the years, the use of guide vanes has been a common strategy for reducing the elbow resistance, typically achieved by modifying the radius or curvature of the vane [148]. Recently, researchers have been exploring alternative methods, including adopting nature-inspired designs such as the sawtooth structures of the leading edges of whale pectoral fins and bat wings, as shown in Figure 23A [148]. The results showed that bionic guide vanes reduced the local resistance coefficient and improved the uniformity of the velocity distribution, with an expanded high-velocity region (in red) and a reduced low-velocity region (in blue) downstream of the elbow, as shown in Figure 23B. It also decreased the turbulence dissipation on the inner elbow surface, reducing the energy loss at the vane’s leading edge. However, the local resistance reduction is influenced by the vane’s dimensions. For example, the difference in the local resistance coefficient between the normal and bionic guide vanes was the smallest when the groove’s dimensionless height was 0.0312 but increased significantly at 0.00625. As the dimensionless height increased further, the reduction in the resistance coefficient became relatively insignificant. Therefore, to apply this design to real-world ventilation ducts, optimum sawtooth dimensions must be determined, which may vary from one duct to another.

**Figure 24:**
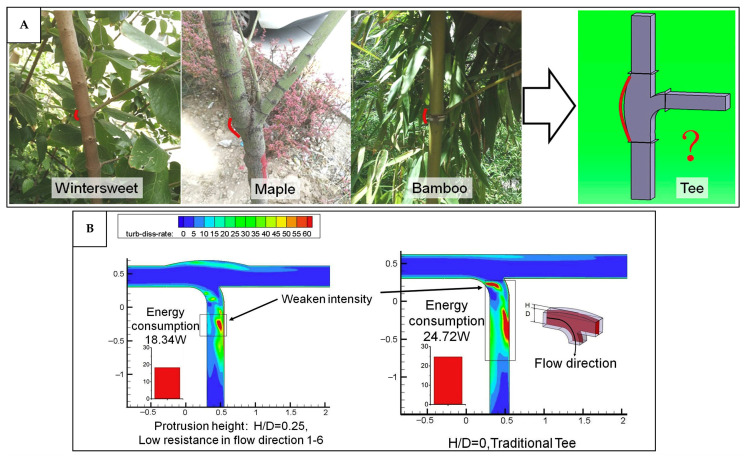
(**A**) Adoption of the tree protrusion feature from various plants for a biomimetic duct tee; (**B**) comparison of turbulent dissipation rates between a traditional (normal) duct tee and a biomimetic duct tee with protrusions. (**A**,**B**) Reproduced with permission from [149].

Another source of high local resistance often occurs at the duct tee. Gao et al. [149] proposed a method for reducing the resistance in the duct tees by incorporating a structure mimicking the shape of joints (protrusions) found in certain plant trunks, as shown in Figure 24A. The results showed that the biomimetic duct tees exhibited a significantly lower resistance than traditional tees in all flow directions, with an average resistance reduction of 22–68% over traditional duct tees. In cases with high local flow rates and low aspect ratios, the resistance became negative, indicating a 100% reduction. They also exhibited smaller dissipation areas and lower energy dissipation rates than the traditional ducts, thereby reducing the energy consumption, as shown in Figure 24B. However, excessive protrusions could be counterproductive, as they induce fluid deformation and increase resistance. Therefore, to achieve maximum resistance reduction, the optimal protrusion height must be determined.

**Figure 25:**
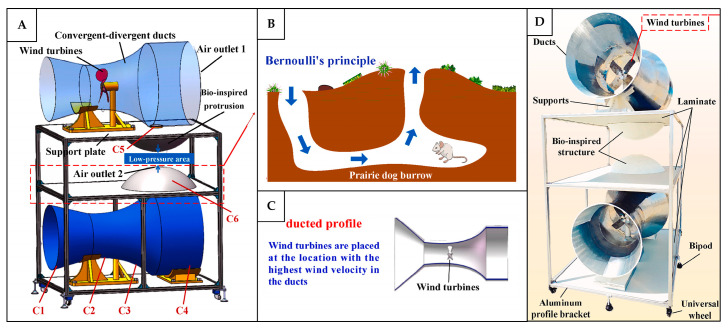
(**A**) Schematic diagram of the prairie-dog-burrow-inspired duct, where C1 denotes the nozzle, C2 the venturi-shaped duct, C3 the diffuser duct, C4 the straight duct, C5 the conduit, and C6 the protrusion. (**B**) The ventilation principle of prairie dog burrows adopted for the bionic duct. (**A**,**B**) Reproduced with permission from [151]. (**C**) Side profile of the bionic duct. Reproduced with permission from [150]. (**D**) Prototype of the bionic duct. Reproduced with permission from [151].

To enhance airflow into a duct, Liu et al. [150,151] proposed a novel duct design (Figure 25) inspired by prairie dog burrows. This bio-inspired design incorporates contraction–expansion sections, venturi-shaped openings, and surface protrusions. The results demonstrated that the protrusions accelerate the airflow locally, generating a low-pressure region that drives the fluid motion from the duct into the conduit and toward the low-pressure zones. Notably, the protrusions reduced the normalized turbulent kinetic energy by approximately 94.5%, while the maximum wind speed within the optimized duct increased by about 107% compared to the conventional duct. Although originally developed for ducted wind turbine applications, this bio-inspired design also has the potential to be applied to the ventilation systems in buildings, where the ducting system plays a crucial role in the air distribution and energy efficiency.

#### 5.1.2. Fan

Noise produced by ventilation fans is a well-known issue in a wide range of indoor environments, from domestic buildings [152] to acoustically sensitive habitats such as the International Space Station [21]. Meanwhile, many organisms in nature have evolved specialized features or structures that reduce noise and improve their aerodynamic performance [153,154,155]. These features share a common characteristic: the presence of non-smooth surface geometries, such as waviness, serrations, grooves, or forked edges, which are commonly found in the trailing and leading edges of owl wings [156,157], the pectoral fins of the humpback whales [158,159,160], the abdomens of the mantis shrimp [161], the surface morphology of the desert scorpion [162], and the shells of various crustaceans [153]. Such designs have been studied in different types of fans, including axial, centrifugal, mixed-flow, and other types of airfoils, ranging from small to large scales. To reduce the noise, these bio-inspired geometries can be applied to different fan components, such as the blade surface, leading and trailing edges, or the blade tips. At the leading edge of the axial fan blades, wavy structures or serrations help reduce the flow resistance and turbulent kinetic energy, suppress boundary layer separation, and consequently decrease noise [163]. However, they can also generate counter-rotating vortices and shift sound sources downstream, which may increase the broadband noise near the trailing edge [164]. When applied to the blade surface, the ridge or textured structures reduce the turbulent kinetic energy and delay the laminar-to-turbulent transition, thereby weakening the vortex formation at the trailing edge and lowering the noise levels [153]. These modifications also enhance the airflow inside the impeller by reducing the resistance and suppressing the boundary layer separation [153]. When incorporated at the trailing edge, serrated structures reduce the noise, but they often do so at the expense of the aerodynamic performance [165].

**Figure 26:**
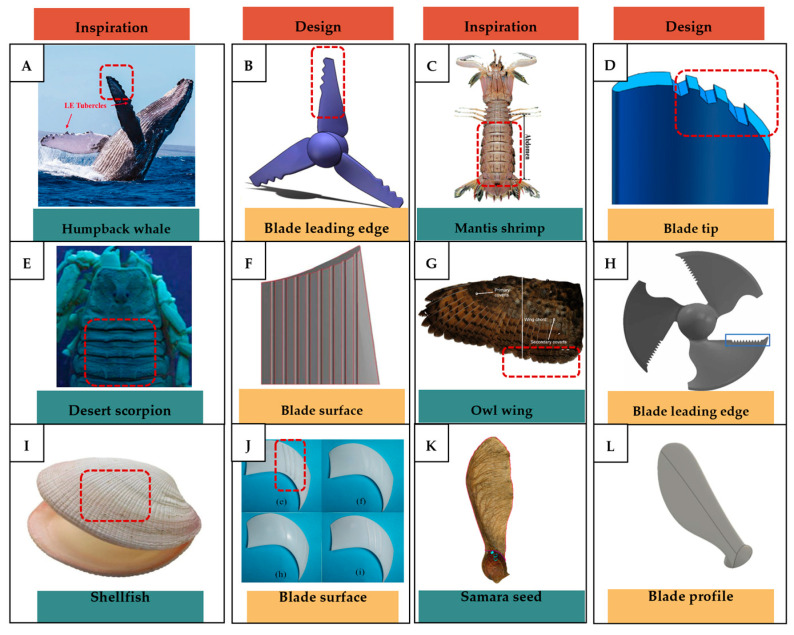
(**A**) Humpback whale pectoral fins. Reproduced with permission from [176], © 2020 Institution of Mechanical Engineers. (**B**) Blade leading edge inspired by the humpback whale. Reproduced with permission from [177]. (**C**) Mantis shrimp abdomen. (**D**) Blade tip inspired by the abdomen of mantis shrimps. (**C**,**D**) Reproduced from [178], CC BY 4.0. (**E**) Desert scorpion. (**F**) Blade surface inspired by the desert scorpion. (**E**,**F**) Reproduced with permission from [162]. (**G**) Owl wing. Reproduced from [179], CC BY 4.0. (**H**) Blade leading edge inspired by owl wings. Reproduced from [163], CC BY 4.0. (**I**) Shellfish. (**J**) Blade surface inspired by shellfish. (**I**,**J**) Reproduced with permission from [153], © 2020 Institution of Mechanical Engineers. (**K**) Samara seed. Reproduced from [180], CC BY-SA 4.0. (**L**) Blade profile inspired by samara seed. Reproduced with permission from [175].

For aerodynamic performance improvement, inspiration has been drawn from butterfly wings, which demonstrate exceptional aerodynamic performance and efficiency due to their effective airflow control, structural flexibility, and precise wing positioning [166,167]. Inspired by this, Tian et al. [168] developed bionic fan blades with depression structures along the blade’s outer edges to reduce the power consumption and enhance the aerodynamic performance of the axial flow fans. The increase in the depression depth initially improves the aerodynamic performance, as evidenced by a higher flow rate and efficiency compared to the baseline design. However, when the depression depth exceeded 1.9 mm, the fan’s efficiency and volumetric flow rate began to decline, while the noise levels increased. Another example is the sycamore seed, whose winged structure enables gliding [169,170], spinning [171,172], and rolling [173,174] during descent. Gururaj et al. [175] applied this principle to ceiling fan blades, finding that the bio-inspired fan delivered slightly lower airflow overall, but it achieved higher air velocity at a greater distance compared to conventional fans. Figure 26 summarizes features and structures observed in various organisms in nature and their application to different types and components of fans.

#### 5.1.3. Envelope

The building envelope, particularly its façade and wall components, plays a critical role in determining the performance of any ventilation system, whether natural, mechanical, or hybrid. This is because it serves as the primary interface separating the exterior and interior environments, directly regulating the exchange of air and moisture between indoor and outdoor spaces. Therefore, enhancing the performance of the building envelope by incorporating responsive or adaptive functionalities can significantly optimize ventilation efficiency within the built environment.

- Humidity (hydro) responsive and adaptive component

**Figure 27:**
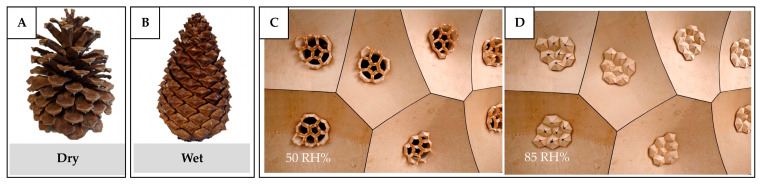
(**A**) Pine cone scales in dry conditions. (**B**) Pine cone scales in wet conditions. (**A**,**B**) Reproduced from [184], CC BY 4.0. (**C**) HygroSkin envelope apertures in the open state (low humidity). (**D**) HygroSkin envelope apertures in the closed state (high humidity). (**C**,**D**) Reproduced with permission from [188].

A robust example of a humidity-responsive and adaptive element in nature is pine cones, which respond to changes in the ambient humidity through reversible movements [181,182,183]. When the environmental humidity is low, i.e., in dry conditions, the pine cones open their scales (Figure 27A) to facilitate seed dispersal, allowing seeds to be carried away by the winds. When the humidity level is high, i.e., in rainy conditions, the scales close (Figure 27B) to protect the seeds, as the wet weather is unfavorable for germination [183,184,185]. Like pinecone scales, the wood composites also exhibit hygroscopic behavior, enabling them to respond to the changes in the humidity levels [185,186,187,188,189]. These composites can be integrated into the building envelopes to function as a component that adapts and responds to the variations in the ambient air humidity [187,188,190]. One of the most prominent examples of this application is the HygroSkin-Meteorosensitive Pavilion, whose envelope contains numerous aperture elements, which can autonomously respond to the changes in humidity levels [191,192]. The apertures were programmed to open (Figure 27C) when the external humidity around the Hygroskin is low and to close when the humidity is high (Figure 27D).

**Figure 28:**
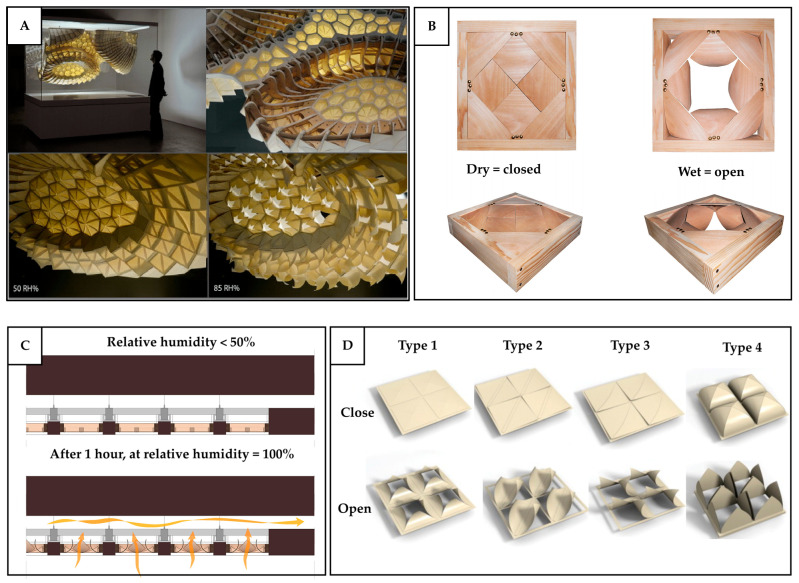
(**A**) Hygroscope installation with humidity-responsive components in open and closed states depending on humidity levels. Reproduced with permission from [188]. (**B**) Un-plywood, closed when dry and open when wet. (**C**) Schematic diagram illustrating the opening and closing of ceiling-mounted ventilation panels in response to humidity levels. (**B**,**C**) Reproduced with permission from [187]. (**D**) Different geometric arrangements of the responsive components in open and closed states. Reproduced with permission from [188].

A similar mechanism, though operating in the opposite manner, is utilized in the HygroScope (Figure 28A) [188]. It consists of rectangular and circular apertures that increase their porosity as the humidity inside the enclosure rises to ventilate out the moisture-saturated air and close themselves when the humidity drops to retain the internal conditions. Apart from the envelope applications, similar components called “un-plywood” have been proposed for the ceiling-mounted ventilation panels [187]. They were programmed to open at relative humidity levels above 50% (wet) and to close below this threshold (Figure 28B,C). For example, as the indoor air becomes warm and humid from occupant activity, the panels autonomously open, allowing the excess heat and moisture to be expelled with the help of the natural convection process.

One of the important factors influencing the overall performance of responsive and adaptive components is the geometric arrangement, which can lead to significant variations in the degree and direction of the opening. For example, when the elements were arranged tangentially to the reference surface (Figure 28D, type 1–3), an increase in the humidity caused them to curl and open through an increased curvature. In contrast, when the reactive elements were rotated out of the surface into a perpendicular orientation (Figure 28D, type 4), an increase in the curvature led to closing, whereas a decrease in the curvature resulted in opening. Therefore, selecting the most suitable patterns or geometric arrangements of the elements is crucial for their application in ventilation systems because the percentage of opening of the responsive elements can significantly influence and regulate how much air can pass through the openings and the level of the humidity required to achieve the desired percentage of opening [188].

**Figure 29:**
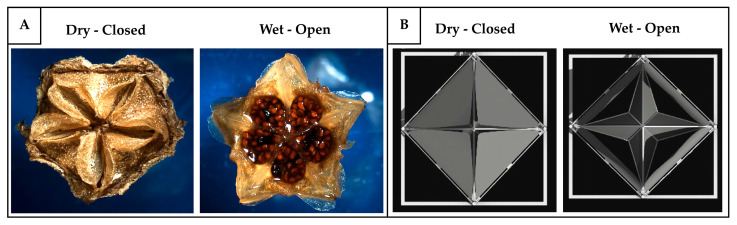
(**A**) Folding (dry) and unfolding (wet) of ice plant seed capsules. Reproduced with permission from [194]. (**B**) Responsive element closed when dry and open when hydrated (wet). Reproduced with permission from [195].

Like pine cones, certain species of ice plants exhibit a sophisticated seed-dispersal mechanism driven by the water-induced folding and unfolding of their seed capsules [193,194,195,196]. These capsules consist of several valves that remain closed under dry conditions (Figure 29A) and autonomously unfold backward when hydrated (Figure 29B) [195]. Inspired by this, Khosromanesh and Asefi [195,196] developed a concept for a hydro-actuated building façade to enhance natural ventilation. The façade system comprises modular square grids with responsive elements, each consisting of four triangular valves connected to a central base. A sunlight sensor is integrated into the system to detect an increase in sunlight and initiate the movement of the responsive elements. The valves and the middle base of the responsive elements consist of honeycomb frameworks filled with hydrogel. When hydrated, the hydrogel swells, causing the elements to open, thus allowing the fresh air to ventilate the building and the used air to be expelled. In the dry state, as the hydrogel loses water, the valves contract and close [194,196]. Figure 29B shows the elements in their open and closed states.

However, like many other bio-inspired designs, the practical applications of the hydro-actuated façades present several challenges. While the opening and closing mechanisms function relatively easily at small model scales, they can become far more complex and difficult to control as the system is scaled up. The precise synchronization of movements across larger surfaces may require more advanced actuation systems, increasing both the design complexity and the risk of malfunction. The use of multiple interconnected and delicate components introduces higher maintenance demands, which in turn increase the operational costs. Therefore, before real-world implementation, considerations such as durability, responsiveness under real weather fluctuations, and long-term cost-effectiveness will be critical in determining whether this design solution can progress from conceptual models to practical applications.

b. Temperature-, humidity- and air-velocity-adaptive components

Mimosa pudica is a highly sensitive plant that exhibits reversible movements, such as drooping its petioles, in response to external stimuli like wind, vibration, or touch [171,181]. Inspired by this, Sankaewthong et al. [197] proposed an innovative façade design called the mimosa kinetic façade to enhance the efficiency and performance of natural ventilation. Similarly to how the mimosa pudica reacts to external stimuli, this bio-inspired façade can respond and adapt to changes in environmental conditions such as temperature, humidity, and air velocity through the use of sensors and motors. The results of their study showed that the mimosa’s kinetic façade enhances the natural ventilation efficiency, improves the quality of indoor air, and promotes better occupant comfort, compared to the traditional static façades. Most of the mimosa’s kinetic façade arrangements show a significant increase in the indoor air quality of up to 12 m/s, compared to the maximum of 2.5 m/s in the single-sided traditional static façade. This kinetic façade also achieves a lower concentration of CO_2_ at only 400 ppm (compared to more than 1000 ppm in a traditional façade) and a maximum of 14.50–19.50 air change per hour (ACH). These results demonstrate the potential of the kinetic façades in improving the quality of the indoor air and the occupants’ comfort. However, further research is required for achieving a balance between the improvement in the indoor air quality and promoting the occupants’ comfort. The future research could also explore the application of such a design in different settings and various climate conditions.

c. Heat-responsive and -adaptive component

**Figure 30:**
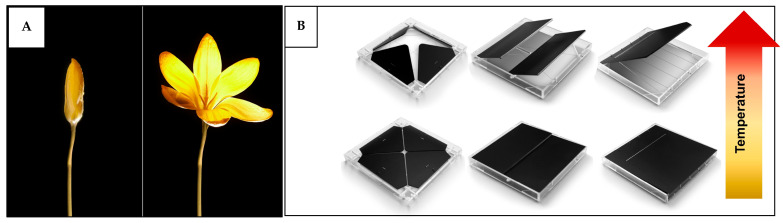
(**A**) Yellow crocus flower (**B**) Crocus flower-inspired ventilation panels in open and closed states, responding to temperature changes. (**A**,**B**) Reproduced with permission from [199].

Crocus flowers (Figure 30A) exhibit a characteristic known as thermonasty, which is a kinetic movement of the plants due to temperature changes. Certain species, such as *Crocus vernus*, can respond to temperature shifts as small as 0.2 °C [198]. The unique heat response of this flower is attributed to the bi-layer structure of its petals, composed of the inner and outer cell layers. At colder temperatures, the outer layer expands faster than the inner layer, causing the petal to bend inward and close. Conversely, at warmer temperatures, the inner layer grows faster, resulting in the petals moving outward to open [199]. Inspired by this, LIFT Architects [199] developed a concept and prototype for thermally active ventilation panels, called the Air Flower (ER) (Figure 30B), which consists of “petals” that incorporate Shape Memory Alloy (SMA) wires as actuating components. When the temperature reaches the SMA’s transformation point, the wires contract, causing the petals to open, and as the temperature decreases and the wires cool, the petals close again. This mechanism can be adapted to a range of building components to provide responsive (adaptive) ventilation, including roof vents, double-skin ventilated façades, and traditional apertures. For example, when the indoor temperature rises to 26 °C, the Air Flower opens its petals to increase the airflow into the building, and once the interior cools, the petals close to seal it. The number and geometry of petals can be customized. For instance, to promote buoyancy-driven natural ventilation generated by the temperature and humidity differences between the indoor and outdoor environments, a simplified version with two rectangular petals can be installed at both the upper and lower openings of a structure. In this configuration, the higher indoor temperature would cause the upper petals to open, allowing the warm air to rise and escape through the top opening, while the cooler, fresh air would enter the building through the petals at the bottom.

### 5.2. System Optimization

In addition to physical designs, nature has also provided algorithmic solutions for the optimization of the ventilation systems as a whole. For example, in underground mines, where the ventilation is energy-intensive and critical for safety, several swarm intelligence and evolutionary algorithms, such as c, ant colony algorithm (ACA), beetle antennae search (BAS), particle swarm optimization (PSO), bare-bones PSO (BBPSO), and multi-strategy BSO (MBSO), have been proposed to reduce the power consumption [200,201,202,203], improve the energy efficiency [201,204], provide ventilation on demand [201], invert the gas explosion source point [205], and stabilize the airflow during the main fan switchovers [206]. Comparative studies highlight their different strengths. For example, Lu et al. [201] showed that the MBSO achieved the greatest reduction in energy consumption, with high accuracy, stable convergence, and improved volumetric flow rates, though at the expense of a longer convergence time. In contrast, the BSO provided the fastest convergence and good optimization but with a lower accuracy, while the BAS and PSO tended to underperform on complex networks due to a lower convergence precision. Another study [202] comparing the genetic algorithms PSO and BBPSO reported that the BBPSO achieved the best balance of convergence accuracy and computational efficiency, making it suitable for the large-scale ventilation systems. Apart from network optimization, the bio-inspired algorithms have also been applied to the fan switchover control [206]. Since coal mines typically operate dual main fans for redundancy, airflow instabilities often occur during fan maintenance changeovers. An improved PSO method, which was tested in a coal mine in China, showed that it reduced the airflow volatility to below 0.4% during the switchover, thereby enhancing both the safety and efficiency of the ventilation system [206]. Similarly, the ACA has been used to optimize the ventilation network configurations, as demonstrated at the Handan Mine Bureau in China [200]. The ACA identified which airways should be retained, excavated, or abandoned, thus lowering the overall ventilation costs.

In building ventilation, nature-inspired algorithms such as the Whale Optimization Algorithm (WOA) and particle swarm optimization (PSO) have been applied to identify the pollutant sources of unknown heights [207]. These algorithms were programmed into source localization codes and executed by mobile robots. Results showed that both algorithms were highly adaptable to the changes in the pollutant source heights. However, the WOA_3D outperformed the PSO_3D, achieving a higher success rate of 77.8% compared to only 55.5% for the PSO_3D, though it required slightly more localization steps. Additionally, the WOA_3D’s random search strategy helped to prevent the robots from getting stuck in the local extremum areas, thus improving its success rate, whereas the PSO_3D was more prone to premature convergence.

### 5.3. Comparative Summary Bio-Inspired Designs and Strategies for Optimizing Ventilation Systems and Components

The comparative analysis of various bio-inspired designs for optimizing ventilation system components indicates that, although such designs generally achieve their intended purposes (e.g., noise reduction or resistance reduction), they often involve certain trade-offs. Table 5 summarizes the key advantages and limitations associated with these approaches, while Table 6 presents a comparative evaluation of their reliability, feasibility, and efficiency across different scales. This comparison highlights the practical challenges and performance variations that must be addressed for the successful implementation of bio-inspired ventilation strategies.

## 6. Applications and Integration of Bio-Inspired Ventilation in Off-Earth Habitats and Underground Mines

### 6.1. Ventilation Challenges in Off-Earth Habitats and Underground Mines

To identify where bio-inspired designs and strategies could enhance the ventilation in hostile environments such as off-Earth habitats and underground mines, it is essential to first understand the challenges faced by current systems. For off-Earth habitats, lessons can be drawn from existing facilities like the International Space Station (ISS) and other previous space missions. Reviews of reports and studies on the ISS, Apollo missions, and the Space Shuttle highlight noise as the primary ventilation issue [18,211,212]. Unlike terrestrial compartments, space habitats are isolated and confined, creating an acoustically challenging environment due to continuous noise sources from the Environmental Control and Life-Support Systems (ECLSSs) [213,214]. Various mitigation measures, such as mufflers, isolators, wraps, covers, and barriers, have been implemented, yet addressing noise at the source through quieter fans remains the most preferable approach [214]. Passive measures, while useful, often become costly over time and consume valuable space needed for essential mission equipment [214,215,216].

Other challenges associated with the ventilation systems in space include the risk of CO_2_ accumulation and poor air distribution. In microgravity, the absence of natural convection increases the likelihood of CO_2_ buildup around an occupant’s head when they remain stationary, as the exhaled air does not disperse as easily as it does on Earth. Continued exposure to the elevated CO_2_ concentrations can impair cognitive function and, in severe cases, cause unconsciousness or even death from asphyxiation [215,217]. Inadequate airflow also contributes to temperature rises in the occupied zones, which is a major cause of crew discomfort [21,211]. While abrasive dust particles have not posed a major issue for the ventilation system aboard the ISS, dust is one of the most significant environmental challenges that astronauts face during lunar or other planetary surface exploration, as has been demonstrated by past lunar missions and is anticipated for future missions to the Moon and Mars. On these planetary surfaces, fine dust particles tend to adhere to astronauts’ spacesuits and equipment. Once brought into the habitat or spacecraft, the dust can become airborne and circulate throughout the cabin, potentially both compromising the equipment such as ventilation systems and posing serious health risks to the crew [218,219].

Unlike off-Earth environments, Earth benefits from the natural forces such as the wind and convection, which are the primary drivers for natural ventilation. However, ventilation systems in underground mines continue to face significant challenges. As the shallow coal and mineral deposits become depleted, mining operations are forced to move to the greater depths [220,221,222,223], which has profound implications for ventilation efficiency and costs. With the increasing depth, the distance that air must travel also increases, leading to higher operating and maintenance costs, as well as greater leakage throughout the system, all of which contribute to rising power consumption [11,224,225]. In addition, deeper mines experience elevated temperatures due to air auto-compression and increased heat transfer from the surrounding rock strata [11,221]. For example, in mines in Germany, the temperature is approximately 40 °C at a depth of 900 m, increasing to about 50 °C at a depth of 1712 m. In one of the deepest underground mines in South Africa, operating at a depth of 3000 m, temperatures can reach up to 70 °C [221]. To counter this heat gain and maintain safe working conditions, substantially higher airflow rates must be supplied. However, the required additional air volume and associated ventilation costs become increasingly dramatic beyond certain depths. For example, in Canadian mines operating at a depth of more than 2000 m, an additional 300 m increase in the depth requires a 20% increase in the air supply, resulting in ventilation costs that are 73% higher in these deeper mines [11].

The growing mechanization of mining, particularly through the use of diesel-powered equipment, introduces further challenges, as the ventilation systems must dilute higher concentrations of toxic emissions [223,226], thereby requiring even greater volume of airflow and energy consumption [11,227]. Mine ventilation systems are also a major contributor to greenhouse gas emissions due to their substantial power consumption and dependence on fossil fuels [227]. Depending on the mine, ventilation may account for 20–40% of the total energy demand and up to 50% of the electricity consumed in the underground operations [228,229]. In the United States, approximately 63% of the mine electricity is supplied by off-grid sources that generate power from carbon-based fuels [230]. This heavy reliance on fossil energy significantly elevates the carbon footprint of mining activities, making the ventilation systems a critical target for energy efficiency improvements and sustainable practices. Lastly, underground mine ventilation systems are often designed to operate continuously at peak capacity, irrespective of the actual demand. While this approach is intended to guarantee the worker’s safety under all possible operating conditions, it also creates significant inefficiencies. For instance, the mines frequently supply air at the maximum volume around the clock, even in situations where it is unnecessary, such as during periods of low activity or when the electric-powered equipment is used. This results in the over-ventilation of the mines and substantial unnecessary energy consumption [11].

### 6.2. Bio-Inspired Solutions for Ventilation in Off-Earth Habitats and Underground Mines

After the challenges stated under Section 6.1 of this paper are identified, the potential applications of the bio-inspired solutions for the ventilation systems in underground mines and space habitats can be explored. These solutions may be integrated into various components of the ventilation systems, including fans, ducts, and air distribution mechanisms. For example, mimicking the environmentally responsive movements of plants could allow the ventilation systems to dynamically adapt to the environmental changes and occupant demands, enhancing human comfort while reducing energy consumption [187,191,197]. This adaptability is particularly valuable for off-Earth habitats, such as future lunar and Martian colonies, where ensuring the crew’s health, as well as comfort, for the mission’s success is crucial and where the energy resources are limited. In underground mines, bio-inspired approaches offer opportunities for ventilation optimization, which is crucial to improving efficiency and reducing energy usage. Table 7 summarizes the relevant bio-inspired solutions to each identified challenge or issue associated with the ventilation of underground mines and off-Earth habitats.

Despite being a promising avenue, the application and integration of bio-inspired approaches in space habitat ventilation remain very limited. These limitations stem from several factors. The interest in bio-inspiration within the research community has surged only in the past decade, and the opportunities to apply such concepts in real missions or habitats have been scarce. Human space missions are still relatively few, and the number of operational habitats in actual space environments has been extremely limited. Integrating such new untested technologies into the already existing systems presents significant challenges, including increased costs, time constraints, and compatibility issues. Although some bio-inspired technologies have been tested on Earth, accurately replicating the effects of microgravity and human factors remains a challenge [231]. In addition, many bio-inspired ventilation strategies developed on Earth also rely on natural forces like natural convection, which do not exist in the microgravity of the off-Earth environment [15,232]. Furthermore, the numerical models used for simulation are often simplified, which may yield promising results for basic designs but raise questions about their feasibility in more complex and intricate designs. Hence, it is important to study the performance of bio-inspired ventilation structures under various settings, including under microgravity conditions.

Likewise, the application of bio-inspired approaches to underground mine ventilation has remained very limited. This could be due to the fact that the ventilation is a critical safety system for both the workers and equipment in the hostile environment of underground mines. Consequently, stakeholders are generally cautious about adopting new designs that have not yet been rigorously validated or demonstrated under real operating conditions. Nevertheless, the research on the applications of nature-inspired algorithms for mine ventilation optimization has grown significantly over the past decade, which has been proposed to improve the energy efficiency, deliver optimum ventilation on demand, invert the gas explosion sources, and stabilize the airflow during fan switchover. For the application of other bio-inspired approaches, it is essential to first simulate and evaluate the designs under a range of climate conditions, as some concepts may be more suitable for specific environments. In addition, developing prototypes and testing them under realistic operating scenarios is crucial to ensuring that the anticipated benefits of the proposed bio-inspired models do not come at the expense of safety.

## 7. Research Gaps and Future Directions

This review has identified several key research gaps and future directions in bio-inspired ventilation research, which can help future researchers to identify the areas that are still lacking and determine what aspects should be prioritized in future studies to address the limitations of current work. The main points are outlined as follows:

- Occupant comfort and indoor air quality (IAQ): Most bio-inspired ventilation studies, particularly those based on passive mechanisms or adaptive components, do not account for occupant comfort and indoor environmental quality, even though these are key requirements of ventilation systems. Future studies should quantify thermal comfort and IAQ by reporting metrics such as predicted mean vote (PMV), predicted percentage of dissatisfied (PPD), draft rate, age of air, humidity, CO_2_, and noise. These metrics can be evaluated in CFD (with coupled heat and contaminant transport) and verified through experiments or full-scale/prototype testing.
- Laboratory or experimental testing coupled with CFD study: A much smaller proportion of studies conducted both experiments and a CFD study rather than CFD-only work. To increase reliability, future research should start with a CFD study for the design of experiments and sensitivity analysis, then reproduce the most optimized case as a prototype for experimental testing.
- Increase the confidence (credibility) of CFD study: While some studies justify their use of assumptions and report grid-independence tests, others lack this. The credibility of the CFD study can be improved by performing and reporting mesh-independence/GCI studies, clearly justifying all assumptions, and validating the CFD result with experimental testing. Where new experiments are not feasible, validation should be carried out against high-quality existing experimental datasets.
- Upscaling. Many experimental studies remain at a small scale, which may not reflect the performance at larger scales. Future research should progress to intermediate and larger scales (even if not yet full scale) to demonstrate consistency across scales and to identify scale-dependent behaviors. A small-scale study should also explicitly apply similarity criteria to ensure that the scaled-down physical models or simulations accurately represent their behavior at full scale.
- Optimization and cost effectiveness: Very few studies explicitly optimize designs or evaluate the cost-effectiveness of the models. More structured designs of experiments and multi-objective optimization should be used to identify balanced solutions and to estimate the costs required to build and operate the prototype.
- Translation to real-world applications: Only a small number of models have been translated from laboratory settings to real-world applications, with bio-inspired fans being a notable exception. Structural limits and scaling challenges can hinder performance in complex real environments. For concepts that are well tested in the lab and after adequate upscaling, the next step can be pilot installation and evaluation in real-world settings if feasible.
- Integration of multiple strategies. Although single strategies are simpler to study and more reliable in general, combining several compatible bio-inspired mechanisms can, in some cases, deliver better performance. Few papers attempt such integration, possibly due to time or scope constraints. Future studies should integrate well-understood strategies and report how such an integration affects airflow, comfort, energy use, and noise.
- Links to space-habitat applications. No studies explicitly consider bio-inspired ventilation for space habitats, despite its clear potential. Future research can investigate the relevant bio-inspired ventilation for space applications, even at the component level, to see how it can improve the habitable environment. In particular, low-noise bio-inspired fans are worth exploring for space habitat applications, where noise can significantly affect occupant comfort and safety. Prototypes can also be tested with lunar dust simulants under relevant environmental conditions to see how quickly parts wear, clog, or degrade.
- Reliability testing. Reliability is rarely assessed, likely because most work is at an early stage. Future research should include reliability and durability testing, such as dust/fouling cycles, thermal/humidity cycling, and prolonged operation to quantify performance stability and the probability of failure over time.
- To better evaluate the efficiency and performance of the bio-inspired ventilation systems and components, certain metrics can be used, which are summarized in Table 8.

## 8. Conclusions

Bio-inspired ventilation offers pathways toward more sustainable and resilient design solutions by minimizing energy consumption, reducing dependence on mechanical systems, and promoting passive regulation of indoor environments. These principles are not only vital for achieving long-term environmental goals on Earth but also hold significant promise for ensuring livable, self-sustaining conditions in extraterrestrial habitats, where efficiency and resource conservation are essential for survival. This study has provided a comprehensive overview of the bio-inspired mechanisms, designs, and strategies found in nature that are relevant to ventilation systems, along with how these concepts have been applied across different settings. By consolidating this knowledge, the review serves both as an entry point for researchers interested in bio-inspired ventilation systems and as a guide for engineers in identifying and applying suitable principles to develop next-generation ventilation systems that are quiet, smart, and energy-efficient through the selection and integration of multiple bio-inspired mechanisms within a unified design.

**Figure A1:**
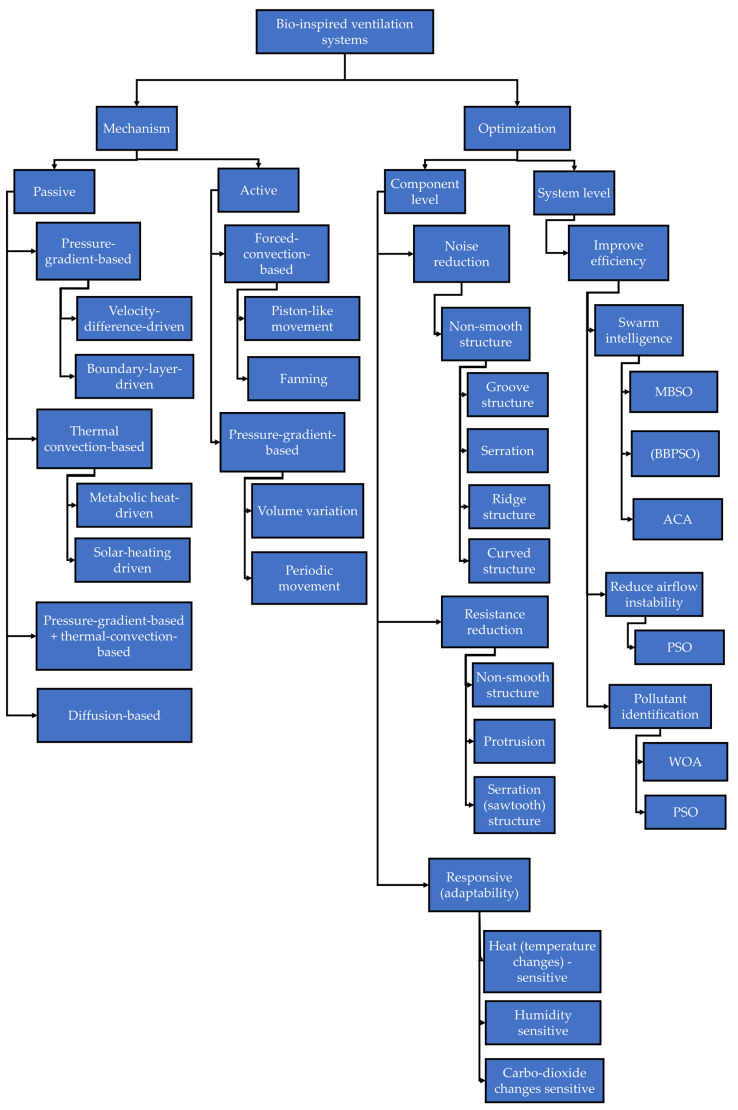
The classification of bio-inspired ventilation based on mechanisms and component optimization.

This review also presents a classification of current bio-inspired ventilation, categorized according to their underlying mechanisms and optimization strategies at both component and system levels, which is shown in Appendix A (Figure A1). Furthermore, it examines the limitations and challenges associated with existing strategies across different applications. By addressing these aspects, this work helps to identify where future research should focus. Despite the increasing research activity in this area, current work remains largely limited to computational studies, with only a few examples providing experimental validation to support computational fluid dynamics (CFD) predictions. Most approaches are isolated, focusing on individual mechanisms or strategies rather than integrated solutions. Moreover, translation to real-world applications remains limited due to scalability, material, and control challenges.

Future research should aim to increase the confidence and reliability of the computational study of bio-inspired ventilation by employing a design of experiments (DoE) approach before progressing to experimental testing. This will help identify the most optimal model configuration while reducing the cost and complexity of physical experiments. In addition, the similarity criteria of the small-scale computational or experimental models should be clearly defined and explicitly justified to demonstrate that the bio-inspired design performs consistently across different scales. For adaptive or responsive components, incorporating smart materials with improved controllability and sensitivity is strongly encouraged to ensure that these systems can respond more effectively and dynamically to changing environmental conditions. For applications characterized by a strong pressure or thermal gradient, passive bio-inspired ventilation mechanisms are recommended, as they can enhance natural ventilation performance with minimal energy use and cost. In contrast, for applications where pressure or temperature gradients are weak, or where occupant comfort is a primary concern, a hybrid approach that combines passive and active mechanisms is encouraged to achieve more stable and controllable ventilation performance.

The application of bio-inspired ventilation in underground mines remains very limited, and for off-Earth habitats, it is virtually non-existent. Introducing untested or unverified designs in such high-risk environments is inherently risky. While these systems may demonstrate effectiveness at a small scale, their application at a larger scale may encounter difficulties due to the complexity of mimicking nature’s designs as well as because of the constraints of underground mines or space missions, such as limited energy resources and harsh environmental conditions. Nevertheless, certain bio-inspired concepts hold clear potential at the component level, particularly for optimizing ventilation fans, where noise reduction is a critical factor in enclosed or space-based habitats. Future research should therefore explore the application of bio-inspired components in analogue space environments and subject them to harsh environmental simulations, for example, by testing fan components exposed to lunar dust simulants, to evaluate material wear and long-term performance under extreme conditions. In addition, unsteady or time-varying ventilation inspired by biological breathing rhythms could be investigated for space-habitat applications, as such approaches may help improve air mixing, contaminant dispersion, and thermal comfort in enclosed environments.

## Figures and Tables

**Table 1:** Keywords for the literature search.

Biomimicry-Related Keywords	Boolean	Subject-Related Keywords
bio-inspired* OR bioinspired* OR biomimetic* OR bioinspiration* OR bio-inspiration* OR nature-inspired* OR nature-based* OR biomimicry* OR bio-design* OR bionic* OR organism-inspired* OR plant-inspired*	AND	vent* OR fan*
		duct*
		air AND regulat*
		wall* OR envelope*
		air AND circulat* HVAC* OR “heating ventilation and air conditioning”

**Table 2:** Inclusion and exclusion criteria.

Criteria for Inclusion	Criteria for Exclusion
Documents published between 2000 and 2025 inclusive	Duplicate studies or multiple reports from the same research with no additional data or insights
Studies on ventilation for the indoor and outdoor environment	Studies lacking bio-inspired designs/strategies
Studies relevant to ventilation systems and their components	Documents written in other languages or containing a significant portion of confusing and unintelligible discussions
Studies considered important for improving the ventilation systems, even if they are not solely focused on ventilation	Pure review studies that do not present new conceptual designs
Written in the English language	Only a small portion of the study is relevant to ventilation systems, or it is not deemed significant for the improvement of ventilation systems.

**Table 3:** The summary of the advantages and limitations of passive ventilation mechanisms across various applications.

Ventilation Mechanism	Implementation Strategy	Applications	Advantages	Challenges/Limitations	Study
Pressure-gradient-based.	Multi-chamber systems connected to occupied spaces and external air.	High-rise buildings.	Improved airflow for the indoor space; enhanced the performance of natural ventilation.	Sensitive to wind availability; performance depends heavily on geometry and weather conditions.	[99]
Pressure-gradient-based.	Asymmetric height, shape, and size of tunnel entrances and exits.	Underground tunnels.	Higher air exchange and flow rate; improved natural ventilation.	Implementation for complex, interconnected tunnel networks is challenging and may be less effective in practice; the effect of urban microclimate is overlooked.	[52]
Pressure-gradient-based.	Strategic building reorientation and rearrangement to generate pressure differences.	Medium to high-rise buildings.	Improved natural ventilation and thermal comfort; reduced cooling load and energy consumption.	Ineffective in extreme cold; unsuitable for buildings with multiple attached blocks.	[106]
Pressure-gradient-based.	Optimum building height and layout to generate pressure differences between buildings.	Medium to high-rise buildings.	Improved pedestrian wind conditions; reduced the static air zones; enhanced the natural ventilation; and decreased the wind pressure on the windward side of the buildings.	Improved wind speed might affect occupant comfort; sensitive to building spacing and surrounding structures.	[107]
Combined pressure-gradient-based and convection-based.	Buildings with chimneys and lower openings, using heat produced by occupants.	High-rise buildings.	Reduced cooling load and reliance on air conditioning; lower energy consumption; cooler indoor temperatures; increased airflow rate and speed.	Application in high-rise buildings still relies on fans; sensitive to wind availability; performance depends heavily on geometry and weather conditions.	[26,35,63,100,101,109,121]
Combined pressure-gradient-based and convection-based.	Central chimney(s) or shaft(s) combined with lower-level ventilation openings, using heat produced by occupants and cool heat sinks.	Underground or buried habitat.	Enhanced passive ventilation; more stable indoor thermal conditions.	Construction complexity; high maintenance; risk of flooding.	[78,80,110,112]
Pressure-gradient-based.	Ventilated façade with upper and lower openings or with random extruded openings.	Low-rise buildings.	Improved airflow speed within the slot; reduced wall surface temperature; reduced cooling load; improved energy saving.	Climate/wind dependency; manufacturing and upscaling challenges of the complex extruded openings; unverified across building types/heights.	[34,114]
Pressure-gradient-based.	Artificial surface conduits or reticulated tunnels that connect the indoor and outdoor environment.	Any type of building.	Improved natural ventilation and cooling of living spaces using wind.	Dependence on wind availability and weather conditions; limited control over airflow may cause drafts and discomfort (in a fully passive system); manufacturing challenges for real-scale buildings.	[63,116]
Diffusion-based mechanism due to concentration gradient	Natural ventilation with a heat recovery system.	Any type of building.	Lower heating load, improved airflow and reduced CO_2_ when coupled with stack ventilation.	Does not meet thermal comfort needs in rooms with single occupancy; performance is influenced by the closing/opening of doors.	[120]

**Table 4:** The summary of the advantages and limitations of active ventilation mechanisms across various applications.

Ventilation Mechanism	Implementation Strategy	Applications	Advantages	Challenges/Limitations	Study
Piston-like-movement-induced forced convection.	Piston-like components capable of performing periodic movements within openings that connect the internal and external environments	Any enclosed space.	Promotes mixing of indoor and outdoor air; can be designed with simple mechanical motion.	Requires actuation and energy input; potential issues with noise, maintenance, and synchronization in large-scale applications; the air mixing might not be significant unless there is another natural force such as wind.	[122,128,130]
Periodic inhalation-exhalation.	Time-periodic ventilation system with single or dual inlets.	Rooms or any enclosed space.	More uniform velocity distribution; reduced pollutants in stagnant zones; higher ventilation efficiency; lower age of air.	Computationally demanding to simulate; impact on occupants’ thermal comfort not investigated; the performance is dependent on the period and amplitude used.	[145]
Volume variation induced pressure gradient.	Biomimetic active ventilation (BAV) modules that separate indoor and outdoor environments.	Rooms or any enclosed space.	Faster air exchange rate between indoor and outdoor environments.	The model assumes advection-dominated transport; natural ventilation is only effective with wind inflow; the impact on occupants’ thermal comfort is not considered.	[131]
Volume variation induced pressure gradient.	Components capable of active breathing or pumping in and out.	Façade with components that inhale and exhale.	Improved permeability of envelope but still controllable; improved air velocity distribution and reduced age of air in optimized case.	Complex to manufacture and operate; the use of piezoelectric wire to generate a pressure difference may be inefficient and costly for large-scale buildings.	[144,146]

**Table 5:** Comparative summary of bio-inspired design and strategies for the optimization of ventilation systems.

Source of Inspiration	Mimicked Features	Applications	Advantages	Challenges/Limitations	Study
Bat wings and whale pectoral fins.	Sawtooth structures.	Guide vanes for ventilation ducts.	Reduced local resistance coefficient; improved uniformity of the velocity distribution; reduced energy dissipation rate.	Resistance reduction depends on duct dimensions; local resistance is based on the average value; the noise effect is not considered.	[148]
Tree branch.	Protrusions.	Ventilation ducts.	Reduced resistance and energy consumption; smaller energy dissipation rate.	Excessive protrusions can cause flow deformation and increase resistance instead of reducing it.	[149,208]
Owl wings, whale fins, mantis shrimp, desert scorpion.	Wavy/sinusoidal/non-smooth structures.	Ventilation fans.	Reduced flow resistance; lower turbulent kinetic energy; reduced noise.	Performance and effectiveness depend on fan types, placement, and component types.	[153,154,155,156,158,160,161,164,209]
Prairie dog burrow geometry.	Elevated entrance and convergent-divergent channels.	Duct with contraction–expansion sections and protrusions.	Increased mass flow rate; accelerated airflow; reduced turbulent kinetic energy.	Structural complexity; protrusions and sudden geometric changes may cause noise, vibration, and flow instability.	[150,151]
Fractal structures.	Self-repeating branching patterns.	Fractal ventilation networks.	Improved airflow and cooling uniformity; enhanced cooling in weakly ventilated areas.	Reduced upward penetration; slower air diffusion and cooling rate.	[210]
Pine cone.	Opening and closing of scales.	Adaptive envelope components for ventilation.	Energy-free operation due to autonomous response to humidity changes; lightweight construction; dynamic environmental adaptation.	Limited user control; reduced sensitivity over time from material fatigue; response time influenced by material thickness and size; performance dependent on geometric shape and arrangement.	[187,188,191,192]
Ice plant.	Opening and closing of its seed capsules.	Adaptive envelope components for ventilation.	Dynamic environmental adaptation; responsive to changing conditions.	Manufacturing/upscaling challenges for large structures; precise synchronization of movements is challenging; risk of malfunction.	[195,196]
Mimosa pudica.	Sensitivity and automatic response to external stimuli.	Kinetic façade that facilitates ventilation.	Enhanced ventilation efficiency; improved indoor air quality and airflow.	Difficult to balance between comfort and air quality.	[197]
Crocus flower.	Opening and closing of petals.	Smart and responsive (adaptive). ventilation panels.	Improved natural ventilation; reduced energy use with smart materials.	Requires maintenance; effectiveness decreases with material fatigue; limited temperature response range.	[199]
Beetle (swarm).	Behavioral patterns.	Algorithm for underground mine ventilation optimization.	Reduced energy use; high accuracy; stable convergence; improved volumetric flow.	Requires a longer time for convergence or optimization.	[201]
Animal social behavior.	Collective behavior, such as the flocking of birds or schooling of fish.	Algorithm for underground mine ventilation optimization.	Balanced convergence accuracy and efficiency; suitable for large-scale ventilation systems.	Complex real mine conditions and sensor errors may reduce the reliability of the algorithm.	[202]
Animal social behavior.	Collective behavior, such as the flocking of birds or schooling of fish.	Optimization of mine fan switchover.	Reduced airflow volatility; improved safety and efficiency during the switchover.	Airflow fluctuations still occur during the switchover when some doors are almost fully closed or open.	[206]
Ant colony.	The foraging mechanism of ant colonies to find the shortest path to food.	Algorithm for underground mine ventilation optimization.	Applicable to new and old mines; reduced ventilation cost; optimizes airway use.	Limited validation in real mines; simplified assumptions.	[200]
Humpback whale behavior.	Hunting behavior.	Algorithm for pollutant source identification.	Higher success rate; can prevent robots from getting stuck in a local extremum area.	Required more localization steps; prone to premature convergence.	[207]

**Table 6:** Comparison of feasibility, reliability, and efficiency of different bio-inspired strategies and designs.

Design/ Strategy	Component/ System	Feasibility	Reliability	Efficiency
Sawtooth structures	Duct elbow	Manufacturing the sawtooth structure requires precision equipment and accurate forming, especially at larger scales.	Reliability tends to increase with the increase in the width of serrations but still largely depends on the installation environment.	Resistance reduction is more significant at a larger scale of dimensionless height of the sawtooth structure.
Protrusion	Duct tee	Easy to fabricate using standard duct manufacturing methods, but precision in the transition angle is important to maintain consistent performance.	Protrusion, regardless of the scales, may face issues with wear and accumulation of dust or debris, potentially affecting long-term performance.	The efficiency of the protrusion tends to increase with scale, but excessive protrusion can cause fluid deformation in certain flow directions.
Non-smooth structures	Fan	The scalability depends on the type of fans and the location the bio-inspired structure is being applied to, but larger fans require more manufacturing cost.	Smaller fans tend to be more reliable because they have been more widely tested compared to larger fans.	The efficiency of the bio-inspired structure is dependent on fan types and the type of non-smooth structure, regardless of the scale.
Contraction–expansion sections and protrusions	Duct.	Relatively costly and difficult to manufacture, particularly as scale increases, since larger sizes lead to higher manufacturing costs.	Dust deposition and erosion can occur in various regions of the duct, particularly along surface irregularities, reducing reliability for long-term operation unless regular maintenance is performed.	The efficiency of the features appears randomly at different scales of protrusion height and length of the contraction–expansion section.
Fractal pattern	Ventilation system network.	The pattern is relatively easy to replicate, particularly at smaller scales with less complex branching patterns.	The intricate branching pattern can be difficult to inspect and maintain, which increases the likelihood of blockage over time, reducing overall system reliability.	At larger-scale implementation, the efficiency may decrease because the flow length and branching complexity increase significantly, leading to greater pressure loss due to bending or obstruction.
Responsive to temperature, humidity and airflow condition	Envelope Element.	Scaling up requires advanced fabrication to synchronize the response of the elements; integration with sensors and actuators increases system complexity and maintenance needs.	The use of complex systems with multiple components and parts reduces reliability since each added element introduces additional potential points of failure.	If the system is perfectly calibrated, it can efficiently regulate multiple environmental parameters at once, though practical implementation remains complex.
Responsive to heat	Envelope element.	Scaling up to large panel sizes requires more material, complicating manufacture and cost; the material must be calibrated to the correct activation temperature for particular building conditions.	Reliability benefits from minimal mechanical and electrical complexity but is limited by material fatigue for long-term use.	Responsive efficiency depends on the activation temperature of the material to the local climate. If the activation temperature is too high or low, ventilation performance may be reduced.
Responsive to humidity	Envelope element.	The length of the responsive element is limited by the commonly available veneer size, hence limiting the scaling-up size.	Large fluctuations in humidity and temperature increase material degradation; uneven wetting across the grain leads to reduced long-term responsiveness.	Thinner material reacts rapidly to humidity changes, but with shorter response duration. Thicker samples respond more slowly, but they maintain deformation for a longer period once activated.
MBSO	System optimization	Perform well on large-scale optimization problems.	High accuracy and stable convergence.	Achieved the greatest reduction but at the expense of a longer convergence time
ACA	System optimization.	Perform well on large-scale optimization problems.	Stable convergence and effective global search ability.	May require more computational resources due to complex path updating
BBPSO	System optimization.	Less suitable for large-scale problems.	High convergence speed but may risk premature convergence.	Fast convergence in early iterations with a good balance between exploration and exploitation
PSO	System optimization.	Performs well in moderate-scale problems.	Fast initial convergence but may get trapped in local optima.	Highly efficient for continuous parameter tuning but less effective in highly nonlinear or multi-modal spaces.
WOA	System optimization.	Strong global search capability and good adaptability.	Moderate accuracy with slower convergence at later stages.	Performs well for complex nonlinear problems but may require parameter tuning to avoid stagnation.

**Table 7:** Bio-inspired solutions for ventilation in underground mines and off-Earth habitats.

Challenges	Context	Relevant Solutions	Integration/Application
Thermal comfort issue—heat	Off-Earth habitats	Temperature-responsive ventilation panels that open or close autonomously with the increase or decrease in temperature levels.	Integrated into habitat walls or panels to regulate airflow and heat autonomously.
Thermal comfort issue—humidity	Off-Earth habitats	Humidity-sensitive components or wall systems that adjust permeability depending on the humidity levels.	Integrated into wall panels or habitat envelopes for passive humidity regulation.
Air mixing issue	Off-Earth habitats	Manipulating the height and shape differences between the inlet and outlet openings so that a greater pressure difference can be generated to increase the airflow; using a time-periodic ventilation supply instead of a steady supply.	Incorporated into the habitat duct layouts or the inlet and outlet openings.
CO_2_ build-up issue	Off-Earth habitats	Ventilation system or components that are responsive to the CO_2_ level.	Sensor-actuator system integrated into adaptive ventilation to detect regions with low CO_2_ concentration and supply air based on demand.
Air mixing issue	Underground mines	Height, size, and shape differences between tunnel entry/exit; venturi-shaped openings at tunnel entrances or exits located at higher elevations; time-periodic ventilation supply.	Integrated into mine tunnel designs and auxiliary ventilation systems to enhance airflow.
Air mixing and power consumption issue	Underground mines	Nature-inspired algorithms to balance ventilation demand, safety, and energy use.	Implemented in real-time mine ventilation control systems or in fan operation systems.
Power consumption issue	Underground mines	Heat recovery ventilation to capture waste heat from the exhaust air and reuse it to warm the intake air in cold regions.	Integrated into mine HVAC and heating systems.
Resistance issue	Underground mines and off-Earth habitats	Duct geometry modifications (protrusions and sawtooth guide vanes).	Applied in junctions, tees, and bends to reduce airflow resistance and save more energy (power).
Noise issue	Underground mines and off-Earth habitats	Sawtooth or non-smooth structures in ventilation fans.	Incorporated into appropriate components and locations of ventilation fans.
Dust issue	Underground mines and off-Earth habitat	Lotus-inspired dust-repellent surface materials.	Applied to duct linings and filter housings to minimize dust accumulation.

**Table 8:** Potential metrics for evaluating the performance and efficiency of bio-inspired ventilation.

System or Component Level	Criteria	Index or Metrics
Ventilation system	Ventilation efficiency and indoor air quality	Air change per hour (ACH); air exchange (ventilation) effectiveness; age of air; nominal time constant $\left(\tau_{n}\right)$; contaminant removal effectiveness (CRE); contaminant concentration distribution; relative humidity.
Ventilation system	Thermal comfort	Draught rate; facial-area speed ratio; predicted mean vote (PMV); predicted percentage of dissatisfied (PPD).
Fan	Aerodynamic and aeroacoustics performance	Volumetric flow rate; pressure fluctuations; sound pressure level (SPL); power consumption; specific fan power (SFP).
Duct	Resistance reduction	Energy dissipation rate; power consumption; local resistance coefficient; velocity distribution; turbulent kinetic energy; pressure drop per length.
Adaptive or responsive component	Responsivity and adaptivity	Response time; energy consumption for components that use mechanical and electrical systems; actuation energy.

## References

[1] González-Torres M., Pérez-Lombard L., Coronel J.F., Maestre I.R., Yan D. (2022). A Review on Buildings Energy Information: Trends, End-Uses, Fuels and Drivers. Energy Rep..

[2] Sara K., Noureddine Z. (2015). A Bio Problem-Solver for Supporting the Design, towards the Optimization of the Energy Efficiency. Proceedings of the 2015 6th International Conference on Modeling, Simulation, and Applied Optimization (ICMSAO).

[3] Li Y., Wang W., Wang Y., Xin Y., He T., Zhao G. (2020). A Review of Studies Involving the Effects of Climate Change on the Energy Consumption for Building Heating and Cooling. Int. J. Environ. Res. Public Health.

[4] Ahmed T., Kumar P., Mottet L. (2021). Natural Ventilation in Warm Climates: The Challenges of Thermal Comfort, Heatwave Resilience and Indoor Air Quality. Renew. Sustain. Energy Rev..

[5] Zhang H., Yang D., Tam V.W.Y., Tao Y., Zhang G., Setunge S., Shi L. (2021). A Critical Review of Combined Natural Ventilation Techniques in Sustainable Buildings. Renew. Sustain. Energy Rev..

[6] McPherson M.J. (1993). Subsurface Ventilation and Environmental Engineering, Mine Ventilation Services.

[7] Wen J., Zuo J., Wang Z., Wen Z., Wang J. (2024). Failure Mechanism Analysis and Support Strength Determination of Deep Coal Mine Roadways—A Case Study. Constr. Build. Mater..

[8] de Villiers D.J., Mathews M.J., Maré P., Kleingeld M., Arndt D. (2019). Evaluating the Impact of Auxiliary Fan Practices on Localised Subsurface Ventilation. Int. J. Min. Sci. Technol..

[9] Wang J., Xiao J., Xue Y., Wen L., Shi D. (2024). Optimization of Airflow Distribution in Mine Ventilation Networks Using the Modified Sooty Tern Optimization Algorithm. Min. Metall. Explor..

[10] Wallace K., Prosser B., Stinnette J.D. (2015). The Practice of Mine Ventilation Engineering. Int. J. Min. Sci. Technol..

[11] Halim A. Ventilation Requirements for Diesel Equipment in Underground Mines–Are We Using the Correct Values. Proceedings of the 16th North American Mine Ventilation Symposium.

[12] Hardcastle S.G., Kocsis C.K. (2004). The Ventilation Challenge. Canadian Institute of Mining, Metallurgy & Petroleum. https://www.researchgate.net/profile/Charles-Kocsis/publication/295574832_The_ventilation_challenge/links/59b2fba70f7e9b37434eac73/The-ventilation-challenge.pdf.

[13] von Einem M., Groll R., Heinicke C. (2022). Computational Modeling of a Ventilation Concept for a Lunar Habitat Laboratory. J. Space Saf. Eng..

[14] Georgescu M.-R., Meslem A., Nastase I., Sandu M., Bode F. (2019). Experimental Study of Carbon Dioxide Accumulation on a Model of the Crew Quarters on the ISS. Proceedings of the 2019 International Conference on Energy and Environment (CIEM).

[15] Georgescu M.R., Meslem A., Nastase I., Tacutu L. (2023). An Alternative Air Distribution Solution for Better Environmental Quality in the ISS Crew Quarters. Int. J. Vent..

[16] James J.T. (2007). The Headache of Carbon Dioxide Exposures. Proceedings of the 37th International Conference on Environmental Systems (ICES).

[17] Son C.H., Zapata J.L., Lin C.-H. (2002). Investigation of Airflow and Accumulation of Carbon Dioxide in the Service Module Crew Quarters. Proceedings of the 32nd International Conference on Environmental Systems (ICES).

[18] Broyan J.L., Borrego M.A., Bahr J.F. (2008). International Space Station USOS Crew Quarters Development. Proceedings of the 38th International Conference on Environmental Systems (ICES).

[19] Georgescu M.-R., Nastase I., Meslem A., Sandu M., Bode F. (2019). Design of a Small-Scale Experimental Model of the International Space Station Crew Quarters for a PIV Flow Field Study. E3S Web of Conferences.

[20] Sandu M., Nastase I., Bode F., Croitoru C., Tacutu L. (2018). Preliminary Study on a Reduced Scaled Model Regarding the Air Diffusion inside a Crew Quarter on Board of the ISS. E3S Web Conf..

[21] Bode F., Nastase I., Croitoru C., Sandu M., Dogeanu A., Ursu I. (2018). Preliminary Numerical Studies for the Improvement of the Ventilation System of the Crew Quarters on Board of the International Space Station. INCAS Bull..

[22] Jernigan M., Gatens R., Perry J., Joshi J. (2018). The next Steps for Environmental Control and Life Support Systems Development for Deep Space Exploration. Proceedings of the 48th International Conference on Environmental Systems (ICES).

[23] Shaw L., Garr J., Gavin L., Hornyak D., Matty C., Ridley A., Salopek M., Toon K. International Space Station as a Testbed for Exploration Environmental Control and Life Support Systems 2021 Status. Proceedings of the 50th International Conference on Environmental Systems (ICES).

[24] Karaarslan-Semiz G. (2022). Education for Sustainable Development in Primary and Secondary Schools: Pedagogical and Practical Approaches for Teachers.

[25] Pawlyn M. (2019). Biomimicry in Architecture.

[26] Khelil S., Zemmouri N. (2019). Biomimetic: A New Strategy for a Passive Sustainable Ventilation System Design in Hot and Arid Regions. Int. J. Environ. Sci. Technol..

[27] Mahmoud E. (2010). Biomimicry: A New Approach to Enhance the Efficiency of Natural Ventilation Systems in Hot Climate. International Seminar Arquitectonics Network, Architecture and Research, Barcelona.

[28] Fu S.C., Zhong X.L., Zhang Y., Lai T.W., Chan K.C., Lee K.Y., Chao C.Y.H. (2020). Bio-Inspired Cooling Technologies and the Applications in Buildings. Energy Build..

[29] Shashwat S., Zingre K.T., Thurairajah N., Kumar D.K., Panicker K., Anand P., Wan M.P. (2023). A Review on Bioinspired Strategies for an Energy-Efficient Built Environment. Energy Build..

[30] An S., Shi B., Jiang M., Fu B., Song C., Tao P., Shang W., Deng T. (2023). Biological and Bioinspired Thermal Energy Regulation and Utilization. Chem. Rev..

[31] Abdullah A., Said I., Ossen D.R. (2018). Applications of Thermoregulation Adaptive Technique of Form in Nature into Architecture: A Review. Int. J. Eng. Technol..

[32] Donthu N., Kumar S., Mukherjee D., Pandey N., Lim W.M. (2021). How to Conduct a Bibliometric Analysis: An Overview and Guidelines. J. Bus. Res..

[33] Canuckguy World Map (BlankMap-World) (2006). Wikimedia Commons. https://commons.wikimedia.org/wiki/File:BlankMap-World.svg.

[34] Paar M.J., Petutschnigg A. (2017). Biomimetic Inspired, Natural Ventilated Façade—A Conceptual Study. J. Facade Des. Eng..

[35] AbdUllah A., Said I.B., Ossen D.R. (2018). Cooling Strategies in The Biological Systems and Termite Mound: The Potential of Emulating Them to Sustainable Architecture and Bionic Engineering. ARPN J. Eng. Appl. Sci..

[36] Kleineidam C., Ernst R., Roces F. (2001). Wind-Induced Ventilation of the Giant Nests of the Leaf-Cutting Ant Atta Vollenweideri. Naturwissenschaften.

[37] Zhao Y., Sun H., Tu D. (2018). Effect of Mechanical Ventilation and Natural Ventilation on Indoor Climates in Urumqi Residential Buildings. Build. Environ..

[38] Ren Z., Fu Y., Dong Y., Zhang P., He X. (2022). Rapid Urbanization and Climate Change Significantly Contribute to Worsening Urban Human Thermal Comfort: A National 183-City, 26-Year Study in China. Urban Clim..

[39] Liu Z., Yu C., Qian Q.K., Huang R., You K., Visscher H., Zhang G. (2023). Incentive Initiatives on Energy-Efficient Renovation of Existing Buildings towards Carbon–Neutral Blueprints in China: Advancements, Challenges and Prospects. Energy Build..

[40] Ding Y., Xu J., Wang X., Cai H., Zhou Z., Sun Y., Shi H. (2021). Propagation of Meteorological to Hydrological Drought for Different Climate Regions in China. J. Environ. Manag..

[41] Tong S., Wen J., Wong N.H., Tan E. (2021). Impact of Façade Design on Indoor Air Temperatures and Cooling Loads in Residential Buildings in the Tropical Climate. Energy Build..

[42] Rahman N.M.A., Haw L.C., Fazlizan A. (2021). A Literature Review of Naturally Ventilated Public Hospital Wards in Tropical Climate Countries for Thermal Comfort and Energy Saving Improvements. Energies.

[43] Sari D.P. (2021). A Review of How Building Mitigates the Urban Heat Island in Indonesia and Tropical Cities. Earth.

[44] Yuan C., Zhu R., Tong S., Mei S., Zhu W. (2022). Impact of Anthropogenic Heat from Air-Conditioning on Air Temperature of Naturally Ventilated Apartments at High-Density Tropical Cities. Energy Build..

[45] Taherian H., Peters R.W. (2023). Advanced Active and Passive Methods in Residential Energy Efficiency. Energies.

[46] Al-Shamkhee D., Al-Aasam A.B., Al-Waeli A.H., Abusaibaa G.Y., Moria H. (2022). Passive Cooling Techniques for Ventilation: An Updated Review. Renew. Energy Environ. Sustain..

[47] Laurini E., De Vita M., De Berardinis P., Friedman A. (2018). Passive Ventilation for Indoor Comfort: A Comparison of Results from Monitoring and Simulation for a Historical Building in a Temperate Climate. Sustainability.

[48] Linden P.F. (1999). The Fluid Mechanics of Natural Ventilation. Annu. Rev. Fluid Mech..

[49] Khanal R., Lei C. (2011). Solar Chimney—A Passive Strategy for Natural Ventilation. Energy Build..

[50] Roetzel A., Tsangrassoulis A., Dietrich U., Busching S. (2010). A Review of Occupant Control on Natural Ventilation. Renew. Sustain. Energy Rev..

[51] Khan N., Su Y., Riffat S.B. (2008). A Review on Wind Driven Ventilation Techniques. Energy Build..

[52] Ali A., Yecko P. (2022). Bio-Inspired Passive Ventilation for Underground Networks. Proceedings of the 11th International Conference on Mathematical Modeling in Physical Sciences.

[53] Vogel S. (1978). Organisms That Capture Currents. Sci. Am..

[54] Vogel S., Ellington C.P., Kilgore D.L. (1973). Wind-Induced Ventilation of the Burrow of the Prairie-Dog, *Cynomys ludovicianus*. J. Comp. Physiol..

[55] Stoeckel J.A., Szoka M., Abdelrahman H.A., Davis J.D., Blersch D.M., Helms B.S. (2021). Crayfish Chimneys Function as Burrow-Ventilation Structures. J. Crustac. Biol..

[56] Korb J., Linsenmair K.E. (2000). Ventilation of Termite Mounds: New Results Require a New Model. Behav. Ecol..

[57] Bignell D.E., Roisin Y., Lo N. (2010). Biology of Termites: A Modern Synthesis.

[58] Worall M. (2011). Homeostasis in Nature: Nest Building Termites and Intelligent Buildings. Intell. Build. Int..

[59] Noirot C., Darlington J.P.E.C., Abe T., Bignell D.E., Higashi M. (2000). Termite Nests: Architecture, Regulation and Defence. Termites: Evolution, Sociality, Symbioses, Ecology.

[60] Darlington J.P. (1984). Two Types of Mound Built by the Termite Macrotermes Subhyalinus in Kenya. Int. J. Trop. Insect Sci..

[61] Geiger R., Aron R.H., Todhunter P. (2009). The Climate near the Ground.

[62] Weir J.S. (1973). Air Flow, Evaporation and Mineral Accumulation in Mounds of Macrotermes Subhyalinus (Rambur). J. Anim. Ecol..

[63] Turner J.S., Soar R.C. (2008). Beyond Biomimicry: What Termites Can Tell Us about Realizing the Living Building. Proceedings of the 1st International Conference on Industrialized, Intelligent Construction (I3CON).

[64] Brickner-Braun I., Zucker-Milwerger D., Braun A., Turner J.S., Pinshow B., Berliner P. (2014). Ventilation of Multi-Entranced Rodent Burrows by Boundary Layer Eddies. J. Exp. Biol..

[65] Turner J.S., Pinshow B. (2015). Transient-State Mechanisms of Wind-Induced Burrow Ventilation. J. Exp. Biol..

[66] Turner J.S. (2000). Architecture and Morphogenesis in the Mound of Macrotermes Michaelseni (Sjöstedt)(Isoptera: Termitidae, Macrotermitinae) in Northern Namibia. Cimbebasia.

[67] White C.R., Seymour R.S. (2021). The Roles of Diffusion and Convection in Ventilation of Animal Burrows. J. Comp. Physiol. B.

[68] Ganot Y., Dragila M.I., Weisbrod N. (2012). Impact of Thermal Convection on Air Circulation in a Mammalian Burrow under Arid Conditions. J. Arid Environ..

[69] Lüscher M. (1961). Air-Conditioned Termite Nests. Sci. Am..

[70] Howe S., Kilgore D.L. (1987). Convective and Diffusive Gas Exchange in Nest Cavities of the Northern Flicker (Colaptes Auratus). Physiol. Zool..

[71] Howe S., Kilgore D.L., Colby C. (1987). Respiratory Gas Concentrations and Temperatures within Nest Cavities of the Northern Flicker (*Colaptes Auratus*). Can. J. Zool..

[72] Roper T.J., Moore J.A.H. (2003). Ventilation of Badger Meles Meles Setts. Mamm. Biol..

[73] Ocko S.A., King H., Andreen D., Bardunias P., Turner J.S., Soar R., Mahadevan L. (2017). Solar-Powered Ventilation of African Termite Mounds. J. Exp. Biol..

[74] King H., Ocko S., Mahadevan L. (2015). Termite Mounds Harness Diurnal Temperature Oscillations for Ventilation. Proc. Natl. Acad. Sci. USA.

[75] Hölldobler B., Wilson E.O. (1990). The Ants.

[76] Jonkman J.C.M. (1980). The External and Internal Structure and Growth of Nests of the Leaf-cutting Ant Atta Vollenweideri Forel, 1893 (Hym.: Formicidae): Part I. Z. Angew. Entomol..

[77] Moreira A., Forti L.C., Andrade A.P., Boaretto M.A., Lopes J. (2004). Nest Architecture of *Atta Laevigata* (F. Smith, 1858) (Hymenoptera: Formicidae). Stud. Neotrop. Fauna Environ..

[78] Bollazzi M., Forti L.C., Roces F. (2012). Ventilation of the Giant Nests of Atta Leaf-Cutting Ants: Does Underground Circulating Air Enter the Fungus Chambers?. Insectes Sociaux.

[79] Bollazzi M., Römer D., Roces F. (2021). Carbon Dioxide Levels and Ventilation in *Acromyrmex* Nests: Significance and Evolution of Architectural Innovations in Leaf-Cutting Ants. R. Soc. Open Sci..

[80] Halboth F., Roces F. (2017). The Construction of Ventilation Turrets in Atta Vollenweideri Leaf-Cutting Ants: Carbon Dioxide Levels in the Nest Tunnels, but Not Airflow or Air Humidity, Influence Turret Structure. PLoS ONE.

[81] Kleineidam C., Roces F. (2000). Carbon Dioxide Concentrations and Nest Ventilation in Nests of the Leaf-Cutting Ant Atta Vollenweideri. Insectes Sociaux.

[82] Quinlan R.J., Cherrett J.M. (1978). Aspects of the Symbiosis of the Leaf-cutting Ant *Acromyrmex Octospinosus* (Reich) and Its Food Fungus. Ecol. Entomol..

[83] Cosarinsky M.I., Roces F. (2012). The Construction of Turrets for Nest Ventilation in the Grass-Cutting Ant Atta Vollenweideri: Import and Assembly of Building Materials. J. Insect Behav..

[84] Weber N.A. (1972). Gardening Ants, the Attines.

[85] Powell R.J., Stradling D.J. (1986). Factors Influencing the Growth of Attamyces Bromatificus, a Symbiont of Attine Ants. Trans. Br. Mycol. Soc..

[86] Cussler E.L. (2009). Diffusion: Mass Transfer in Fluid Systems.

[87] Chen Y., Wang J., Flanagan D.R. (2017). Fundamental of Diffusion and Dissolution. Developing Solid Oral Dosage Forms.

[88] Pillow J.J. (2005). High-Frequency Oscillatory Ventilation: Mechanisms of Gas Exchange and Lung Mechanics. Crit. Care Med..

[89] Haddad M., Sharma S. (2023). Physiology, Lung.

[90] Petersson J., Glenny R.W. (2014). Gas Exchange and Ventilation–Perfusion Relationships in the Lung. Eur. Respir. J..

[91] Pallone T.L., Turner M.R., Edwards A., Jamison R.L. (2003). Countercurrent Exchange in the Renal Medulla. Am. J. Physiol.-Regul. Integr. Comp. Physiol..

[92] Imai M., Taniguchi J., Tabei K. (1987). Function of Thin Loops of Henle. Kidney Int..

[93] Mount D.B. (2014). Thick Ascending Limb of the Loop of Henle. Clin. J. Am. Soc. Nephrol..

[94] Hetherington A.M., Woodward F.I. (2003). The Role of Stomata in Sensing and Driving Environmental Change. Nature.

[95] Lawson T., Vialet-Chabrand S. (2019). Speedy Stomata, Photosynthesis and Plant Water Use Efficiency. New Phytol..

[96] Lawson T., Von Caemmerer S., Baroli I., Lüttge U.E., Beyschlag W., Büdel B., Francis D. (2010). Photosynthesis and Stomatal Behaviour. Progress in Botany 72.

[97] Doheny-Adams T., Hunt L., Franks P.J., Beerling D.J., Gray J.E. (2012). Genetic Manipulation of Stomatal Density Influences Stomatal Size, Plant Growth and Tolerance to Restricted Water Supply across a Growth Carbon Dioxide Gradient. Philos. Trans. R. Soc. B Biol. Sci..

[98] Franks P.J., Beerling D.J. (2009). Maximum Leaf Conductance Driven by CO_2_ Effects on Stomatal Size and Density over Geologic Time. Proc. Natl. Acad. Sci. USA.

[99] Wei Y., Lin Z., Wang Y., Wang X. (2023). Simulation and Optimization Study on the Ventilation Performance of High-Rise Buildings Inspired by the White Termite Mound Chamber Structure. Biomimetics.

[100] Verbrugghe N., Rubinacci E., Khan A.Z. (2023). Biomimicry in Architecture: A Review of Definitions, Case Studies, and Design Methods. Biomimetics.

[101] Yuan Y., Yu X., Yang X., Xiao Y., Xiang B., Wang Y. (2017). Bionic Building Energy Efficiency and Bionic Green Architecture: A Review. Renew. Sustain. Energy Rev..

[102] Claggett N., Surovek A., Capehart W., Shahbazi K. (2018). Termite Mounds: Bioinspired Examination of the Role of Material and Environment in Multifunctional Structural Forms. J. Struct. Eng..

[103] Yahaya Andrew N., Nanlop Uwa J., Odion A. (2023). Passive Cooling Techniques in Historical Building Versus Contemporary Bio Mimic Concepts: An Overview. Am. J. Civ. Eng. Archit..

[104] Brazier D. Eastgate Centre, Harare, Zimbabwe. Wikimedia Commons 2008. https://commons.wikimedia.org/wiki/File:Eastgate_Centre,_Harare,_Zimbabwe.jpg.

[105] ElDin N.N., Abdou A., ElGawad I.A. (2016). Biomimetic Potentials for Building Envelope Adaptation in Egypt. Procedia Environ. Sci..

[106] Waheeb M.I., Hemeida F.A., Mohamed A.F. (2024). Improving Thermal Comfort Using Biomimicry in the Urban Residential Districts in New Aswan City, Egypt. Environ. Dev. Sustain..

[107] Wei Y., He W., Zhang S., Wang X., Peng Y. (2023). CFD Simulation and Optimization of Ventilation for the Layout of Community Architecture Inspired by Fishbone Form. Int. J. Model. Simul. Sci. Comput..

[108] Hou Y. (2018). Effect of Wind Speed on Human Thermal Sensation and Thermal Comfort. Proceedings of the Materials Science, Energy Technology and Power Engineering II (MEP2018).

[109] Ramasam N.G., Kaviarasu V., Adarsh V., Rohithraman R. (2024). Termite Mould Building—A Comparative Study on Bio-Memetics Model. Proceedings of the Advancements in Materials for Civil Engineering Applications.

[110] Nývlt V., Musílek J., Čejka J., Stopka O. (2016). The Study of Derinkuyu Underground City in Cappadocia Located in Pyroclastic Rock Materials. Procedia Eng..

[111] Tsui E., Wei H.D., Hua Z.L., Jie S., Su B., Tao H.J. (2010). The Global Health Community (Termite’s Nest-Based Apartment Complex Design) Guangzhou, China. World Arch..

[112] Yang G., Zhou W., Qu W., Yao W., Zhu P., Xu J. (2022). A Review of Ant Nests and Their Implications for Architecture. Buildings.

[113] Caldwell L.M. (2018). Nature Carved Rock Cappadocia. Wikimedia Commons. https://commons.wikimedia.org/wiki/File:Nature_carved_rock_Cappadocia_03.jpg.

[114] Alyahya A., Lannon S., Jabi W. (2025). Biomimetic Opaque Ventilated Façade for Low-Rise Buildings in Hot Arid Climate. Buildings.

[115] Lee J., Lee H. (2018). Pneumatic Skin with Adaptive Openings—Adaptive Façade with Opening Control Integrated with CFD for Natural Ventilation. Proceedings of the 23rd International Conference on Computer-Aided Architectural Design Research in Asia: Learning, Prototyping and Adapting (CAADRIA).

[116] Andréen D., Soar R. (2023). Termite-Inspired Metamaterials for Flow-Active Building Envelopes. Front. Mater..

[117] Cuce P.M., Riffat S. (2015). A Comprehensive Review of Heat Recovery Systems for Building Applications. Renew. Sustain. Energy Rev..

[118] Kragh J., Rose J., Nielsen T.R., Svendsen S. (2007). New Counter Flow Heat Exchanger Designed for Ventilation Systems in Cold Climates. Energy Build..

[119] Zender–Świercz E. (2021). A Review of Heat Recovery in Ventilation. Energies.

[120] Adamu Z., Price A. (2015). Natural Ventilation with Heat Recovery: A Biomimetic Concept. Buildings.

[121] Alleyne M. (2013). The Termite Mound: A Not-Quite-True Popular Bioinspiration Story. https://insectsdiditfirst.com/2013/09/18/the-termite-mound-a-not-quite-true-popular-bioinspiration-story/.

[122] Ar A., Barnea A., Yom-Tov Y., Mersten-Katz C. (2004). Woodpecker Cavity Aeration: A Predictive Model. Respir. Physiol. Neurobiol..

[123] Ar A., Piontkewitz Y. (1992). Nest Ventilation Explains Gas Composition in the Nest-Chamber of the European Bee-Eater. Respir. Physiol..

[124] Peters J.M., Gravish N., Combes S.A. (2017). Wings as Impellers: Honey Bees Co-Opt Flight System to Induce Nest Ventilation and Disperse Pheromones. J. Exp. Biol..

[125] Simpson J. (1961). Nest Climate Regulation in Honey Bee Colonies: Honey Bees Control Their Domestic Environment by Methods Based on Their Habit of Clustering Together. Science.

[126] Southwick E.E., Moritz R.F. (1987). Social Control of Air Ventilation in Colonies of Honey Bees, Apis Mellifera. J. Insect Physiol..

[127] Moritz R.F.A., Crewe R.M. (1988). Air Ventilation in Nests of Two African Stingless bees Trigona Denoiti and Trigona Gribodoi. Experientia.

[128] Kastberger G., Waddoup D., Weihmann F., Hoetzl T. (2016). Evidence for Ventilation through Collective Respiratory Movements in Giant Honeybee (Apis Dorsata) Nests. PLoS ONE.

[129] Meikle W.G., Barg A., Weiss M. (2022). Honey Bee Colonies Maintain CO_2_ and Temperature Regimes in Spite of Change in Hive Ventilation Characteristics. Apidologie.

[130] Peters J.M., Peleg O., Mahadevan L. (2019). Collective Ventilation in Honeybee Nests. J. R. Soc. Interface.

[131] Marom G., Grossbard S., Bodek M., Neuman E., Elad D. (2023). Computational Analysis of a New Biomimetic Active Ventilation Paradigm for Indoor Spaces. Int. J. Numer. Methods Heat Fluid Flow.

[132] Lessa T.B., de Abreu D.K., Bertassoli B.M., Ambrósio C.E. (2016). Diaphragm: A Vital Respiratory Muscle in Mammals. Ann. Anat.-Anat. Anz..

[133] Kocjan J., Adamek M., Gzik-Zroska B., Czyżewski D., Rydel M. (2017). Network of Breathing. Multifunctional Role of the Diaphragm: A Review. Adv. Respir. Med..

[134] Nelson N. (2012). Diaphragmatic Breathing: The Foundation of Core Stability. Strength Cond. J..

[135] Maish M.S. (2010). The Diaphragm. Surg. Clin..

[136] Wallden M. (2017). The Diaphragm–More than an Inspired Design. J. Bodyw. Mov. Ther..

[137] Klabunde R. (2011). Cardiovascular Physiology Concepts.

[138] Levick J.R. (2013). An Introduction to Cardiovascular Physiology.

[139] Pollock J.D., Makaryus A.N. (2022). Physiology, Cardiac Cycle. StatPearls.

[140] Fukuta H., Little W.C. (2008). The Cardiac Cycle and the Physiologic Basis of Left Ventricular Contraction, Ejection, Relaxation, and Filling. Heart Fail. Clin..

[141] Carlsson M., Cain P., Holmqvist C., Stahlberg F., Lundback S., Arheden H. (2004). Total Heart Volume Variation throughout the Cardiac Cycle in Humans. Am. J. Physiol.-Heart Circ. Physiol..

[142] Chan-Dewar F. (2012). The Cardiac Cycle. Anaesth. Intensive Care Med..

[143] United Nations Environment Programme, World Conservation Monitoring Centre, World Wide Fund for Nature (2010). Deep-Sea Sponge Grounds: Reservoirs of Biodiversity.

[144] Badarnah L., Knaack U. Bio-Inspired Ventilating System for Building Envelopes. Proceedings of the International Conference of 21st Century: Building Stock Activation.

[145] Zhang H.-L., Li B., Shang J., Wang W.-W., Zhao F.-Y. (2023). Airborne Pollutant Removal Effectiveness and Hidden Pollutant Source Identification of Bionic Ventilation Systems: Direct and Inverse CFD Demonstrations. Indoor Air.

[146] Badarnah L., Kadri U., Knaack U. A Bio-Inspired Ventilating Envelope Optimized by Air-Flow Simulations. Proceedings of the 2008 World Sustainable Building Conference (SB08).

[147] Wang H., Li X., Tang Y., Chen X., Shen H., Cao X., Gao H. (2022). Simulation and Experimental Study on the Elbow Pressure Loss of Large Air Duct with Different Internal Guide Vanes. Build. Serv. Eng. Res. Technol..

[148] Zhang C., Li A., Che J., Li Y., Liu Q., Zhao Y. (2022). A Low-Resistance Elbow with a Bionic Sawtooth Guide Vane in Ventilation and Air Conditioning Systems. Build. Simul..

[149] Gao R., Liu K., Li A., Fang Z., Yang Z., Cong B. (2018). Biomimetic Duct Tee for Reducing the Local Resistance of a Ventilation and Air-Conditioning System. Build. Environ..

[150] Liu S., Zhang L., Lu J., Liu Z., Jing Z., Zhang X., Cui X., Wang H. (2026). A Novel Bio-Inspired Ducted Wind Turbine: From Prairie Dog Burrow Architecture to Aerodynamic Performance Optimization. Renew. Energy.

[151] Liu S., Zhang L., Jiao L., Lu J., Liu Z., Jing Z., Zhang X., Cui X., Wang H. (2025). Optimized Empty Duct Geometry for Ducted Wind Turbines: A Prairie Dog Burrow-Inspired Approach. Energy.

[152] Harvie-Clark J., Conlan N., Wei W., Siddall M. (2019). How Loud Is Too Loud? Noise from Domestic Mechanical Ventilation Systems. Int. J. Vent..

[153] Wang S., Yu X., Shen L., Yang A., Chen E., Fieldhouse J., Barton D., Kosarieh S. (2021). Noise Reduction of Automobile Cooling Fan Based on Bio-Inspired Design. Proc. Inst. Mech. Eng. Part J. Automob. Eng..

[154] Lei J., Cui Q., Qin G. (2023). Performance Improvement and Noise Reduction Analysis of Multi-Blade Centrifugal Fan Imitating Long-Eared Owl Wing Surface. Phys. Fluids.

[155] Han Z., Mu Z., Yin W., Li W., Niu S., Zhang J., Ren L. (2016). Biomimetic Multifunctional Surfaces Inspired from Animals. Adv. Colloid Interface Sci..

[156] Jaworski J.W., Peake N. (2020). Aeroacoustics of Silent Owl Flight. Annu. Rev. Fluid Mech..

[157] Wang Y., Zhao K., Lu X.-Y., Song Y.-B., Bennett G.J. (2019). Bio-Inspired Aerodynamic Noise Control: A Bibliographic Review. Appl. Sci..

[158] Fish F.E., Weber P.W., Murray M.M., Howle L.E. (2011). The Tubercles on Humpback Whales’ Flippers: Application of Bio-Inspired Technology. Integr. Comp. Biol..

[159] Miklosovic D.S., Murray M.M., Howle L.E., Fish F.E. (2004). Leading-Edge Tubercles Delay Stall on Humpback Whale (*Megaptera Novaeangliae*) Flippers. Phys. Fluids.

[160] Watts P., Fish F.E. (2001). The Influence of Passive, Leading Edge Tubercles on Wing Performance. Proceedings of the 12th International Symposium on Unmanned Untethered Submersible Technology.

[161] Gu Y., Xia K., Zhang W., Mou J., Wu D., Liu W., Xu M., Zhou P., Ren Y. (2021). Airfoil Profile Surface Drag Reduction Characteristics Based on the Structure of the Mantis Shrimp Abdominal Segment. Arch. Appl. Mech..

[162] Zhang J., Han Z., Yin W., Wang H., Ge C., Jiang J. (2013). Numerical Experiment of the Solid Particle Erosion of Bionic Configuration Blade of Centrifugal Fan. Acta Metall. Sin. Engl. Lett..

[163] Sun Y., Li R., Wang L., Liu C., Yang Z., Ma F. (2024). Bionic Noise Reduction Design of Axial Fan Impeller. J. Phys. Appl. Phys..

[164] Tieghi L., Czwielong F., Barnabei V.F., Ocker C., Delibra G., Becker S., Corsini A. (2024). Aerodynamics and Aeroacoustics of Leading Edge Serration in Low-Speed Axial Fans with Forward Skewed Blades. J. Eng. Gas Turbines Power.

[165] Ye X., Zheng N., Zhang R., Li C. (2022). Effect of Serrated Trailing-Edge Blades on Aerodynamic Noise of an Axial Fan. J. Mech. Sci. Technol..

[166] Le Roy C., Debat V., Llaurens V. (2019). Adaptive Evolution of Butterfly Wing Shape: From Morphology to Behaviour. Biol. Rev..

[167] Zhang Y., Wang X., Wang S., Huang W., Weng Q. (2021). Kinematic and Aerodynamic Investigation of the Butterfly in Forward Free Flight for the Butterfly-Inspired Flapping Wing Air Vehicle. Appl. Sci..

[168] Tian C., Liu X., Wang J., Xi G. (2022). Effects of Bionic Blades Inspired by the Butterfly Wing on the Aerodynamic Performance and Noise of the Axial Flow Fan Used in Air Conditioner. Int. J. Refrig..

[169] Minami S., Azuma A. (2003). Various Flying Modes of Wind-Dispersal Seeds. J. Theor. Biol..

[170] Azuma A., Okuno Y. (1987). Flight of a Samara, Alsomitra Macrocarpa. J. Theor. Biol..

[171] Volkov A.G., Foster J.C., Ashby T.A., Walker R.K., Johnson J.A., Markin V.S. (2010). *Mimosa Pudica*: Electrical and Mechanical Stimulation of Plant Movements. Plant Cell Environ..

[172] Ladera C.L., Pineda P.A. (2009). The Physics of the Spectacular Flight of the Triplaris Samaras. Lat.-Am. J. Phys. Educ..

[173] Green D.S. (1980). The Terminal Velocity and Dispersal of Spinning Samaras. Am. J. Bot..

[174] McCutchen C.W. (1977). The Spinning Rotation of Ash and Tulip Tree Samaras. Science.

[175] Gururaj F., Vijayakumar N., Satish J., Madhusudhana H.K., Aakash C., Siddhi R. (2024). Design and Analysis of Nature-Inspired Design for Ceiling Fan Inspired by Sycamore Seeds to Ensure a More Optimal Air Flow. Proceedings of the 4th International Conference on Advances in Physical Sciences and Materials (ICAPSM).

[176] Su L., Qiang X., Zheng T., Teng J. (2020). Effect of Undulating Blades on Highly Loaded Compressor Cascade Performance. Proc. Inst. Mech. Eng. Part J. Power Energy.

[177] Fan M., Dong X., Li Z., Sun Z., Feng L. (2022). Numerical and Experimental Study on Flow Separation Control of Airfoils with Various Leading-Edge Tubercles. Ocean Eng..

[178] Xu Z., Liu X., Liu Y., Qin W., Xi G. (2022). Flow Control Mechanism of Blade Tip Bionic Grooves and Their Influence on Aerodynamic Performance and Noise of Multi-Blade Centrifugal Fan. Energies.

[179] Weger M., Wagner H. (2016). Morphological Variations of Leading-Edge Serrations in Owls (Strigiformes). PLoS ONE.

[180] Jung B.K., Rezgui D. (2023). Sectional Leading Edge Vortex Lift and Drag Coefficients of Autorotating Samaras. Aerospace.

[181] Ahamed M.K., Wang H., Hazell P.J. (2022). From Biology to Biomimicry: Using Nature to Build Better Structures—A Review. Constr. Build. Mater..

[182] Dawson C., Vincent J.F., Rocca A.-M. (1997). How Pine Cones Open. Nature.

[183] Reyssat E., Mahadevan L. (2009). Hygromorphs: From Pine Cones to Biomimetic Bilayers. J. R. Soc. Interface.

[184] Quan H., Pirosa A., Yang W., Ritchie R.O., Meyers M.A. (2021). Hydration-Induced Reversible Deformation of the Pine Cone. Acta Biomater..

[185] Zhan T., Li R., Liu Z., Peng H., Lyu J. (2023). From Adaptive Plant Materials toward Hygro-Actuated Wooden Building Systems: A Review. Constr. Build. Mater..

[186] Correa D., Papadopoulou A., Guberan C., Jhaveri N., Reichert S., Menges A., Tibbits S. (2015). 3D-Printed Wood: Programming Hygroscopic Material Transformations. 3D Print. Addit. Manuf..

[187] Pelliccia G., Baldinelli G., Bianconi F., Filippucci M., Fioravanti M., Goli G., Rotili A., Togni M. (2020). Characterisation of Wood Hygromorphic Panels for Relative Humidity Passive Control. J. Build. Eng..

[188] Reichert S., Menges A., Correa D. (2015). Meteorosensitive Architecture: Biomimetic Building Skins Based on Materially Embedded and Hygroscopically Enabled Responsiveness. Comput.-Aided Des..

[189] Vailati C., Hass P., Burgert I., Rüggeberg M. (2017). Upscaling of Wood Bilayers: Design Principles for Controlling Shape Change and Increasing Moisture Change Rate. Mater. Struct..

[190] López M., Rubio R., Martín S., Croxford B. (2017). How Plants Inspire Façades. From Plants to Architecture: Biomimetic Principles for the Development of Adaptive Architectural Envelopes. Renew. Sustain. Energy Rev..

[191] Krieg O.D. (2016). HygroSkin–Meteorosensitive Pavilion. Advancing Wood Architecture.

[192] Menges A., Reichert S. (2015). Performative Wood: Physically Programming the Responsive Architecture of the *HygroScope* and HygroSkin Projects. Archit. Des..

[193] Guiducci L., Razghandi K., Bertinetti L., Turcaud S., Rüggeberg M., Weaver J.C., Fratzl P., Burgert I., Dunlop J.W.C. (2016). Honeycomb Actuators Inspired by the Unfolding of Ice Plant Seed Capsules. PLoS ONE.

[194] Harrington M.J., Razghandi K., Ditsch F., Guiducci L., Rueggeberg M., Dunlop J.W.C., Fratzl P., Neinhuis C., Burgert I. (2011). Origami-like Unfolding of Hydro-Actuated Ice Plant Seed Capsules. Nat. Commun..

[195] Khosromanesh R., Asefi M. (2019). Form-Finding Mechanism Derived from Plant Movement in Response to Environmental Conditions for Building Envelopes. Sustain. Cities Soc..

[196] Khosromanesh R., Asefi M. (2022). Towards an Implementation of Bio-Inspired Hydro-Actuated Building Façade. Intell. Build. Int..

[197] Sankaewthong S., Miyata K., Horanont T., Xie H., Karnjana J. (2023). Mimosa Kinetic Façade: Bio-Inspired Ventilation Leveraging the Mimosa Pudica Mechanism for Enhanced Indoor Air Quality. Biomimetics.

[198] Andrews F.M. (1929). The Effect of Temperature on Flowers. Plant Physiol..

[199] Payne A. (2007). The Air Flower (ER). LIFT Archit. https://www.liftarchitects.com/air-flower.

[200] Wei G. (2011). Optimization of Mine Ventilation System Based on Bionics Algorithm. Procedia Eng..

[201] Lu F., Wang K., Wang J., Wang Z., Ma Y. (2025). Research on Optimization and Regulation of Air Volume in Mine Ventilation Network Based on Multi-Strategy Beetle Swarm Optimization. Adv. Eng. Inform..

[202] Li J., Li Y., Zhang W., Dong J., Cui Y. (2022). Multi-Objective Intelligent Decision and Linkage Control Algorithm for Mine Ventilation. Energies.

[203] Babu V.R., Maity T., Burman S. (2016). Energy Saving Possibilities of Mine Ventilation Fan Using Particle Swarm Optimization. Proceedings of the 2016 International Conference on Electrical, Electronics, and Optimization Techniques (ICEEOT).

[204] Babu V.R., Maity T., Burman S. (2016). Optimization of Energy Use for Mine Ventilation Fan with Variable Speed Drive. Proceedings of the 2016 International Conference on Intelligent Control Power and Instrumentation (ICICPI).

[205] Lei B., Zhao C., He B., Wu B. (2021). A Study on Source Identification of Gas Explosion in Coal Mines Based on Gas Concentration. Fuel.

[206] Ge H., Xu G., Huang J., Ma X. (2019). A Mine Main Fans Switchover System with Lower Air Flow Volatility Based on Improved Particle Swarm Optimization Algorithm. Adv. Mech. Eng..

[207] Chen A., Liao Y., Cai H., Guo X., Zhang B., Lin B., Zhang W., Wei L., Tong Y. (2023). Experimental Study on 3D Source Localization in Indoor Environments with Weak Airflow Based on Two Bionic Swarm Intelligence Algorithms. Build. Environ..

[208] Gao R., Fang Z., Li A., Liu K., Yang Z., Cong B. (2018). A Novel Low-Resistance Tee of Ventilation and Air Conditioning Duct Based on Energy Dissipation Control. Appl. Therm. Eng..

[209] Zhang J.-Q., Han Z.-W., Cao H.-N., Yin W., Niu S.-C., Wang H.-Y. (2013). Numerical Analysis of Erosion Caused by Biomimetic Axial Fan Blade. Adv. Mater. Sci. Eng..

[210] Lin J., Chen K., Liu W., Lu X. (2024). Analysis of Flow Field and Temperature Distribution in Granary under Novel Ventilation Systems Based on Fractal Structure. Therm. Sci..

[211] Berville C., Georgescu M.-R., Năstase I. (2019). Numerical Study of the Air Distribution in the Crew Quarters on Board of the International Space Station. E3S Web Conf..

[212] Bode F., Nastase I., Croitoru C.V., Sandu M., Dogeanu A. (2018). A Numerical Analysis of the Air Distribution System for the Ventilation of the Crew Quarters on Board of the International Space Station. E3S Web Conf..

[213] Allen C.S. (2015). International Space Station Acoustics-A Status Report. Proceedings of the 45th International Conference on Environmental Systems (ICES).

[214] Goodman J.R., Grosveld F.W. Acoustics and Noise Control in Space Crew Compartments. 2015, JSC-CN-34152. https://ntrs.nasa.gov/api/citations/20160000818/downloads/20160000818.pdf.

[215] Broyan J., Welsh D., Cady S. (2010). International Space Station Crew Quarters Ventilation and Acoustic Design Implementation. Proceedings of the 40th International Conference on Environmental Systems.

[216] Koch L.D., Stephens D., Goodman J.M., Mirhashemi A., Buerhle R., Sutliff D.L., Allen C.S. (2024). Spacecraft Cabin Ventilation Fan Research at NASA. Proceedings of the 30th AIAA/CEAS Aeroacoustics Conference.

[217] Law J., Watkins S., Alexander D. (2010). In-Flight Carbon Dioxide Exposures and Related Symptoms: Association, Susceptibility, and Operational Implications. NASA Tech. Pap..

[218] Wagner S., Thomas G., Laws B., Lyndon B. (2004). An Assessment of Dust Effects on Planetary Surface Systems to Support Exploration Requirements.

[219] Kobrick R.L., Agui J.H. (2019). Preparing for Planetary Surface Exploration by Measuring Habitat Dust Intrusion with Filter Tests during an Analogue Mars Mission. Acta Astronaut..

[220] He M.C., Guo P.Y. (2013). Deep Rock Mass Thermodynamic Effect and Temperature Control Measures. Chin. J. Rock Mech. Eng..

[221] Nie X., Wei X., Li X., Lu C. (2018). Heat Treatment and Ventilation Optimization in a Deep Mine. Adv. Civ. Eng..

[222] Ranjith P.G., Zhao J., Ju M., De Silva R.V., Rathnaweera T.D., Bandara A.K. (2017). Opportunities and Challenges in Deep Mining: A Brief Review. Engineering.

[223] Demirel N., Awuah-Offei K. (2018). Energy-Efficient Mine Ventilation Practices. Energy Efficiency in the Minerals Industry.

[224] Costa L.d.V., Silva J.M.d. (2020). Strategies Used to Control the Costs of Underground Ventilation in Some Brazilian Mines. REM-Int. Eng. J..

[225] Kamyar A., Aminossadati S.M., Leonardi C., Sasmito A. Current Developments and Challenges of Underground Mine Ventilation and Cooling Methods. Proceedings of the 2016 Coal Operators’ Conference.

[226] Acuña E.I., Lowndes I.S. (2014). A Review of Primary Mine Ventilation System Optimization. Interfaces.

[227] Pouresmaieli M., Ataei M., Qarahasanlou A.N., Barabadi A. (2023). Integration of Renewable Energy and Sustainable Development with Strategic Planning in the Mining Industry. Results Eng..

[228] De Souza E. (2018). Cost Saving Strategies in Mine Ventilation. CIM J..

[229] De Vilhena Costa L., Margarida Da Silva J. (2020). Cost-Saving Electrical Energy Consumption in Underground Ventilation by the Use of Ventilation on Demand. Min. Technol..

[230] Palchak D., Cochran J., Deshmukh R., Ehlen A., Soonee R., Narasimhan S., Joshi M., McBennett B., Milligan M., Sreedharan P. (2017). Greening the Grid: Pathways to Integrate 175 Gigawatts of Renewable Energy into India’s Electric Grid, Vol. I—National Study. https://escholarship.org/uc/item/91n393jd.

[231] Stockman B., Boyle J., Bacon J. (2010). International Space Station Systems Engineering Case Study.

[232] Georgescu M.R., Meslem A., Nastase I. (2020). Accumulation and Spatial Distribution of CO_2_ in the Astronaut’s Crew Quarters on the International Space Station. Build. Environ..

